# Supplementary material for: Multifunctional nanoparticle potentiates the in situ vaccination effect of radiation therapy and enhances response to immune checkpoint blockade
Source: Nat Commun. 2022 Aug 23;13:4948. doi: 10.1038/s41467-022-32645-x (PMC9399096; doi:10.1038/s41467-022-32645-x)
Supplement: Supplementary file 1 — Supplementary Information [file 41467_2022_32645_MOESM1_ESM.pdf]

Supplementary Information

Multifunctional nanoparticle potentiates the in situ vaccination effect of radiation therapy and enhances response to immune checkpoint blockade

Ying Zhang[1,2#], Raghava N. Sriramaneni[3#], Paul A. Clark[3], Justin C. Jagodinsky[3], Mingzhou Ye[1,2], Wonjong Jin[3], Yuyuan Wang[1,2], Amber Bates[3], Caroline P. Kerr[3,4], Trang Le[5], Raad Allawi[3], Xiuxiu Wang[1,2], Ruosen Xie[1,2], Thomas C. Havighurst[5], Ishan Chakravarty[3], Alexander L. Rakhmilevich[3], Kathleen A. O'Leary[6], Linda A. Schuler[6], Paul M. Sondel[3,7], Kyungmann Kim[5], Shaoqin Gong[1,2]*, Zachary S. Morris[3]*

[1]Department of Biomedical Engineering and Wisconsin Institute for Discovery, University of Wisconsin-Madison, Madison, WI, USA
[2]Department of Ophthalmology and Visual Sciences, University of Wisconsin-Madison, Madison, WI, USA
[3]Department of Human Oncology, University of Wisconsin-Madison, Madison, WI, USA
[4]Department of Radiology, University of Wisconsin-Madison, Madison, WI, USA
[5]Department of Biostatistics and Medical Informatics, University of Wisconsin-Madison, Madison, WI, USA
[6]Department of Comparative Biosciences, University of Wisconsin-Madison, Madison, WI, USA
[7]Department of Pediatrics, University of Wisconsin-Madison, Madison, WI, USA
[#] These authors contribute equally.
*Corresponding authors: shaoqingong@wisc.edu; zmorris@humonc.wisc.edu

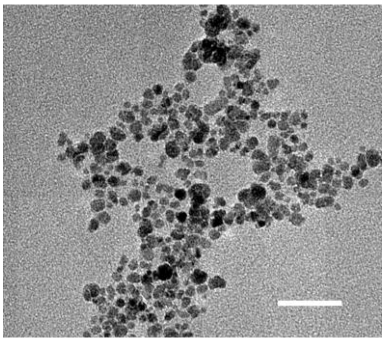

Supplementary Figure 1. A TEM image of the ION. Scale bar: 50 nm. A representative image from three independent samples is shown.

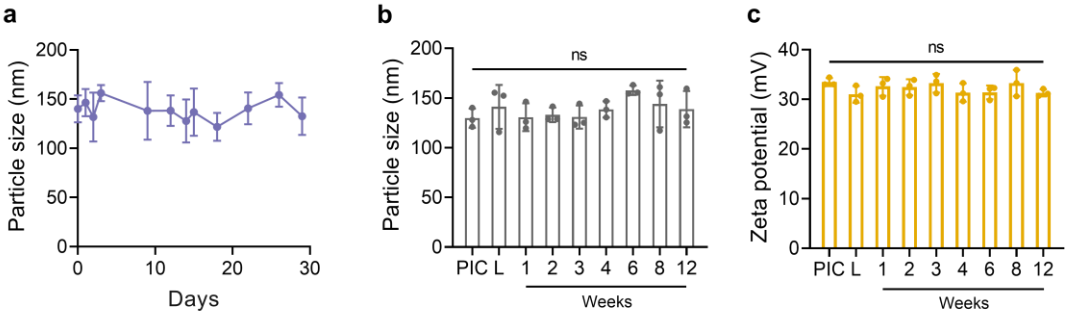

Supplementary Figure 2. (**a**) The particle size of PIC in the presence of 1mM PBS during storage at 4°C. The (**b**) particle size and (**c**) zeta potential of lyophilized PIC in the presence of 1% sucrose during storage for 12 weeks at -20°C. (n=3 independent samples). L: PIC after lyophilization. Data are shown as mean ± SD. Statistical significance was calculated via one-way ANOVA test in **b**-**c**. *p<0.05, **p<0.01, ***p<0.001 and ****p<0.0001. Source data are provided in Source Data file.

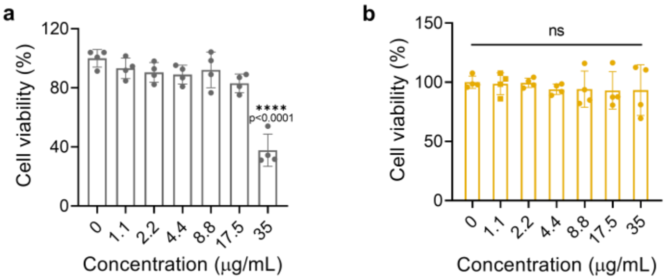

Supplementary Figure 3. The cell viability of (**a**) B78 cells and (**b**) RAW264.7 cells after in vitro co-culture with indicated concentrations of PIC for 48 h. (n=4 biologically independent samples). Data are shown as mean ± SD. Statistical significance was calculated via one-way ANOVA test. *p<0.05, **p<0.01, ***p<0.001 and ****p<0.0001. Source data are provided in Source Data file.

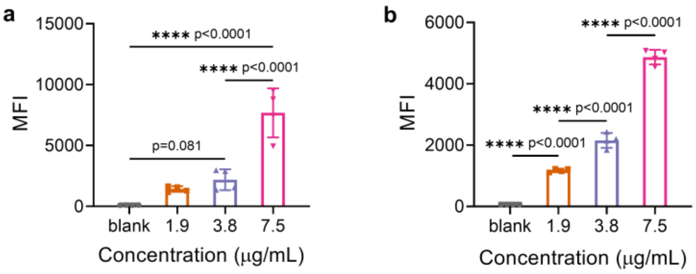

Supplementary Figure 4. The mean fluorescence intensity (MFI) of FITC in (**a**) B78 cells and (**b**) RAW264.7 cells after in vitro treatment with indicated concentrations of FITC-labeled PIC for 2 h. (n=4 biologically independent samples). Data are shown as mean ± SD. Statistical significance was calculated via one-way ANOVA test. *p<0.05, **p<0.01, ***p<0.001 and ****p<0.0001. Source data are provided in Source Data file.

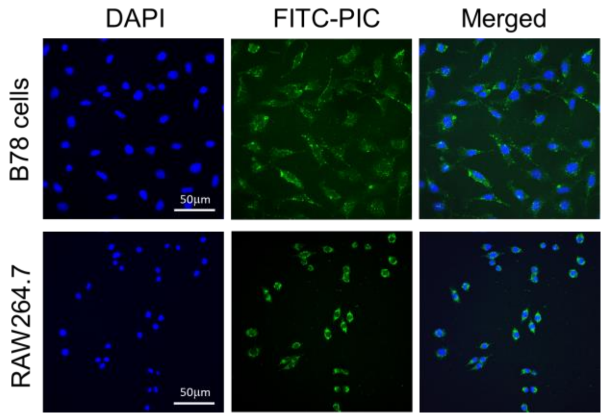

Supplementary Figure 5. Confocal laser scanning microscopy (CLSM) images of B78 cells and RAW264.7 cells after treatment with FITC-labeled PIC for 2h. A representative image of three independent samples from each group is shown.

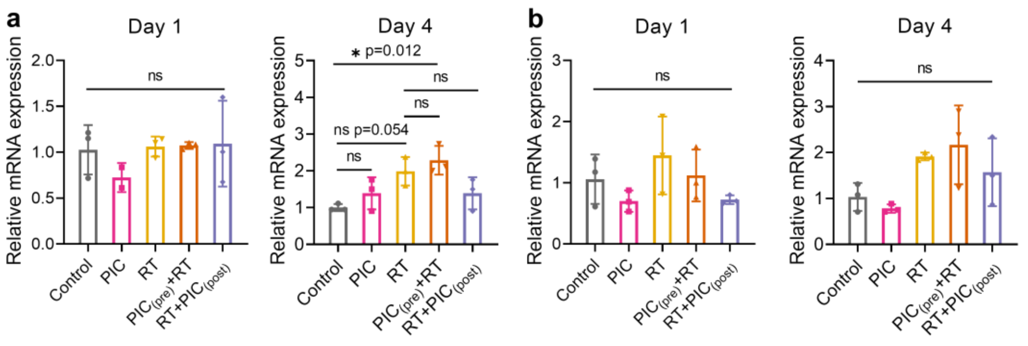

Supplementary Figure 6. The mRNA expression of (**a**) *Ifnβ1* and (**b**) *Pd-l1* in B78 cells at day 1 and day 4

after indicated in vitro treatments. (n=3 biologically independent samples). PIC: 4.67 μg/mL; RT: 12 Gy. The treatments of the cells were given per **Figure 2a**. Data are shown as mean ± SD. Statistical significance was calculated via one-way ANOVA test. *p<0.05, **p<0.01, ***p<0.001 and ****p<0.0001. Source data are provided in Source Data file.

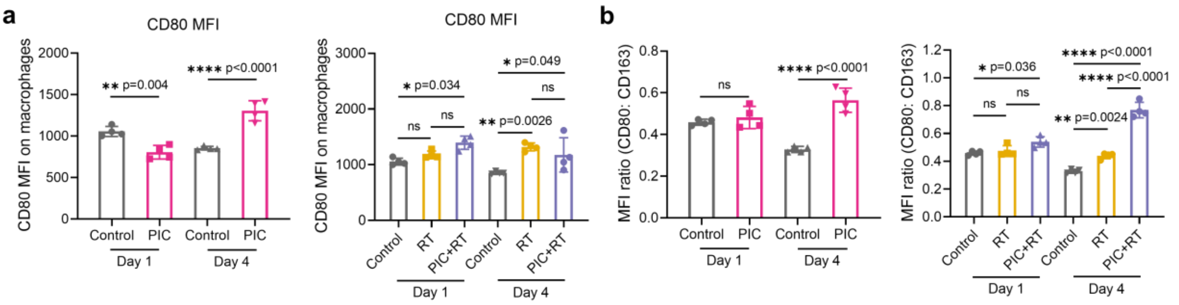

Supplementary Figure 7. The (**a**) mean fluorescence intensity (MFI) of CD80 and (**b**) ratios of CD80 MFI to CD163 MFI on CD11b$^+$F4/80$^+$ BMDMs at day 1 and day 4 after indicated treatments. RT: 12 Gy. PIC: 4.67 μg/mL. The treatments of the cells were given per **Figure 2d**. (n=4 biologically independent samples). Data are shown as mean ± SD. Statistical significance was calculated via one-way ANOVA test. *p<0.05, **p<0.01, ***p<0.001 and ****p<0.0001. Source data are provided in Source Data file.

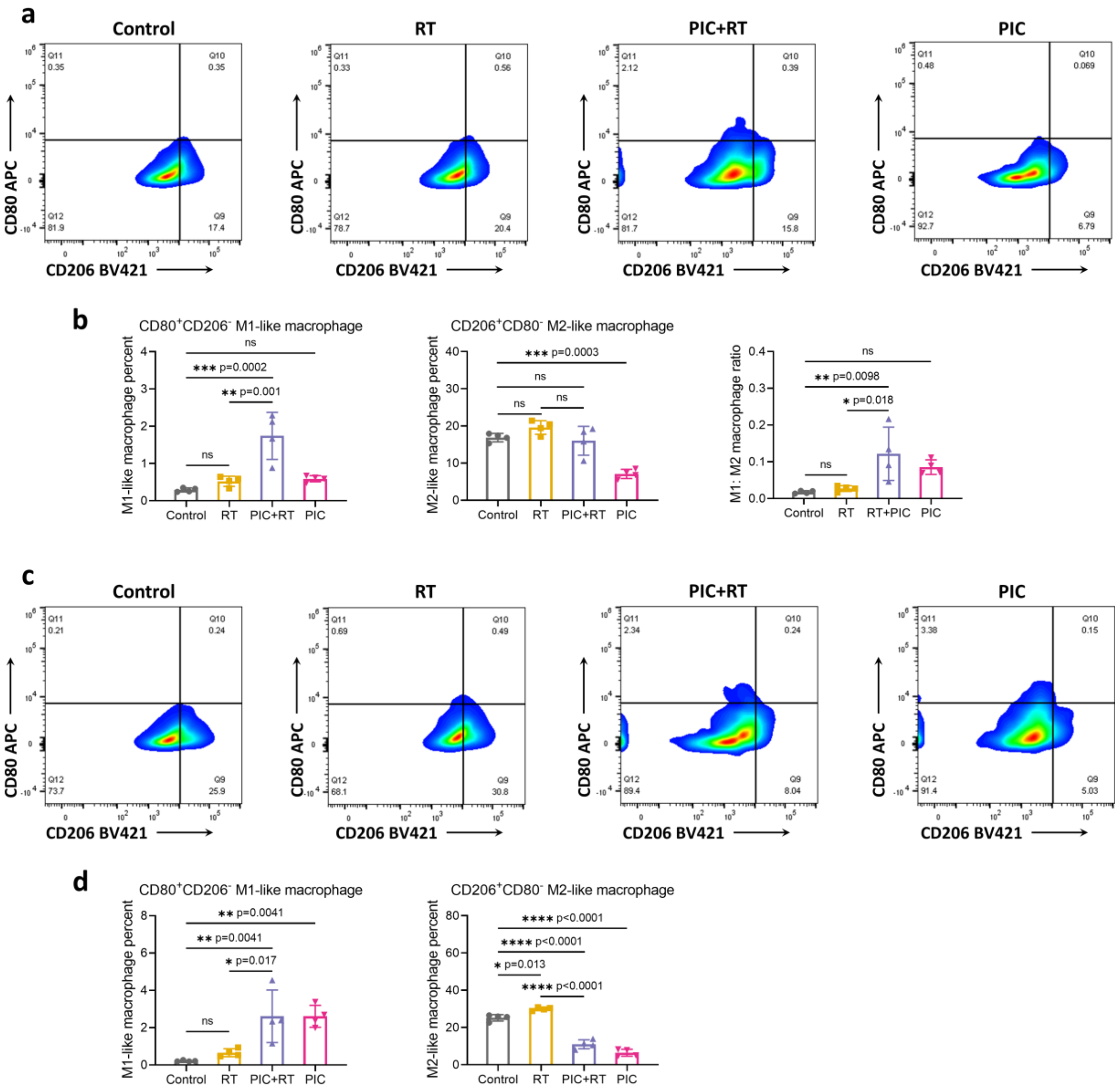

Supplementary Figure 8. (**a**) Representative flow cytometry data and (**b**) quantification of M1-like macrophages ($CD80^+CD206^-$) and M2-like macrophages ($CD206^+CD80^-$) among $CD11b^+F4/80^+$ BMDMs and their ratios at day 1 after indicated treatments. (**c**) Representative flow cytometry data and (**d**) quantification of the M1-like macrophages ($CD80^+CD206^-$) and M2 macrophages ($CD206^+CD80^-$) among $CD11b^+F4/80^+$ BMDMs at day 4 after indicated treatments. (n=4 biologically independent samples). PIC: 4.67 μg/mL; RT: 12 Gy. The treatments of the cells were given per **Figure 2d**. Statistical significance was calculated via one-way ANOVA test in **b** and **d**, and data are shown as mean ± SD. *p<0.05, **p<0.01, ***p<0.001 and ****p<0.0001. Source data are provided in Source Data file.

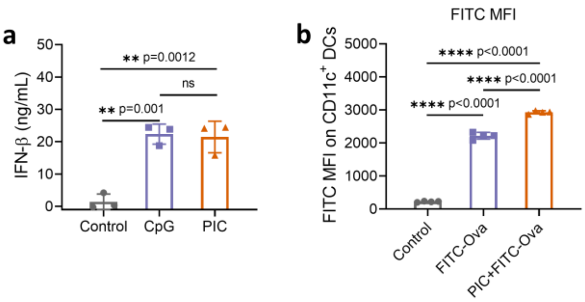

Supplementary Figure 9. (**a**) The concentration of IFN-β secreted from CD11c+ dendritic cells after indicated treatment for 24 hours. CpG: 0.5 μg/mL; PIC: 4.67 μg/mL. (n=3 biologically independent samples). (**b**) The mean fluorescence intensity (MFI) of FITC-Ova in CD11c+ DCs at 24 h after treatment. FITC-Ova: 1.67 μg/mL; PIC: 4.67 μg/mL. (n=4 biologically independent samples). Data are shown as mean ± SD. Statistical significance was calculated via one-way ANOVA test. *p<0.05, **p<0.01, ***p<0.001 and ****p<0.0001. Source data are provided in Source Data file.

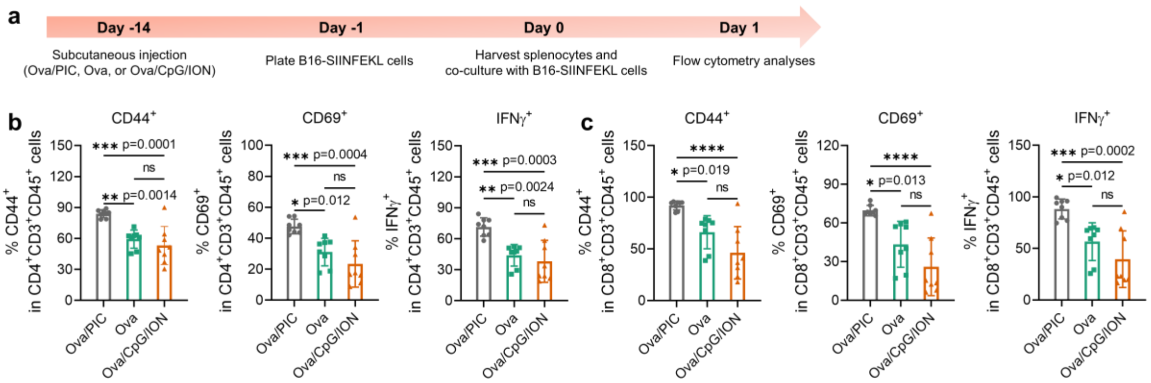

Supplementary Figure 10. (**a**) Scheme for the co-culture of B16-SIINFEKL cells with splenocytes extracted from Ova, Ova/PIC, or Ova/CpG/ION injected mice. Quantification of CD44+, CD69+ and IFNγ+ cells out of (**b**) CD4+CD3+CD45+ and (**c**) CD8+CD3+CD45+ cells in splenocytes by flow cytometry. (n=8 biologically independent samples). Data are shown as mean ± SD. Statistical significance was calculated via one-way ANOVA test. *p<0.05, **p<0.01, ***p<0.001 and ****p<0.0001. Gating strategy is shown in Supplementary Figure 11. Source data are provided in Source Data file.

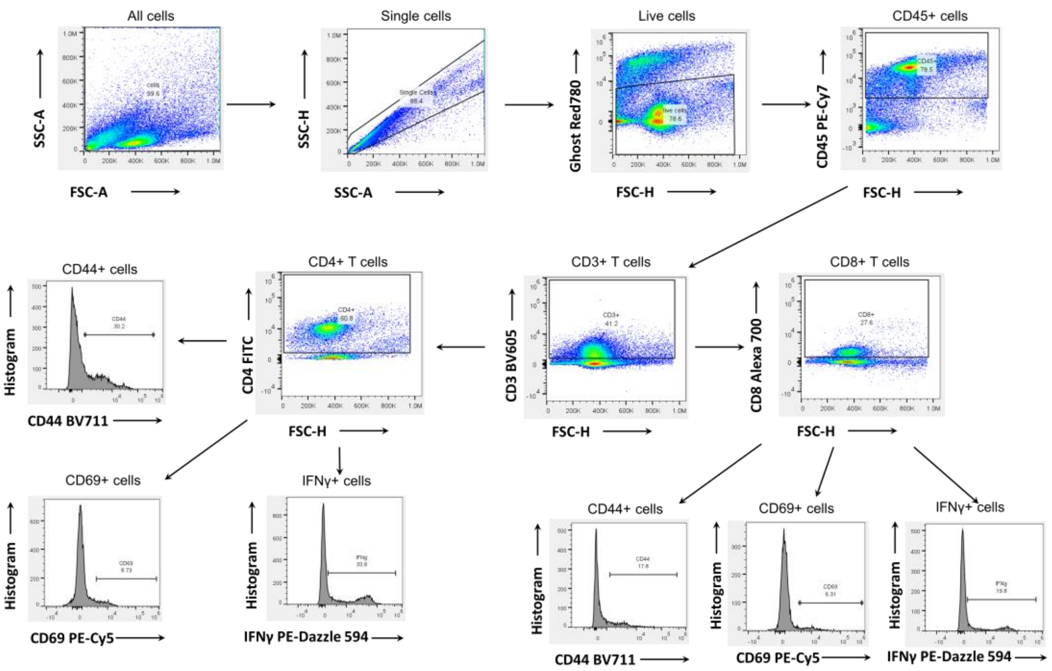

Supplementary Figure 11. Gating strategy of the T cell analysis as shown in Supplementary Figure 10.

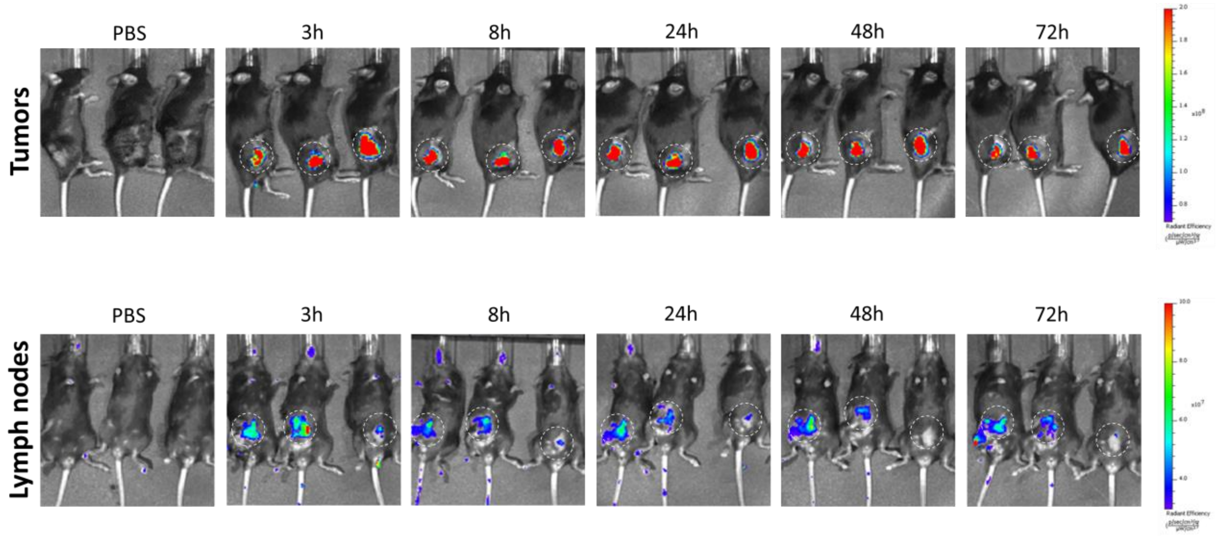

Supplementary Figure 12. IVIS images of B78 melanoma-bearing mice at indicated timepoints after Cy5-labeled PIC (Cy5-PIC) was intratumorally injected.

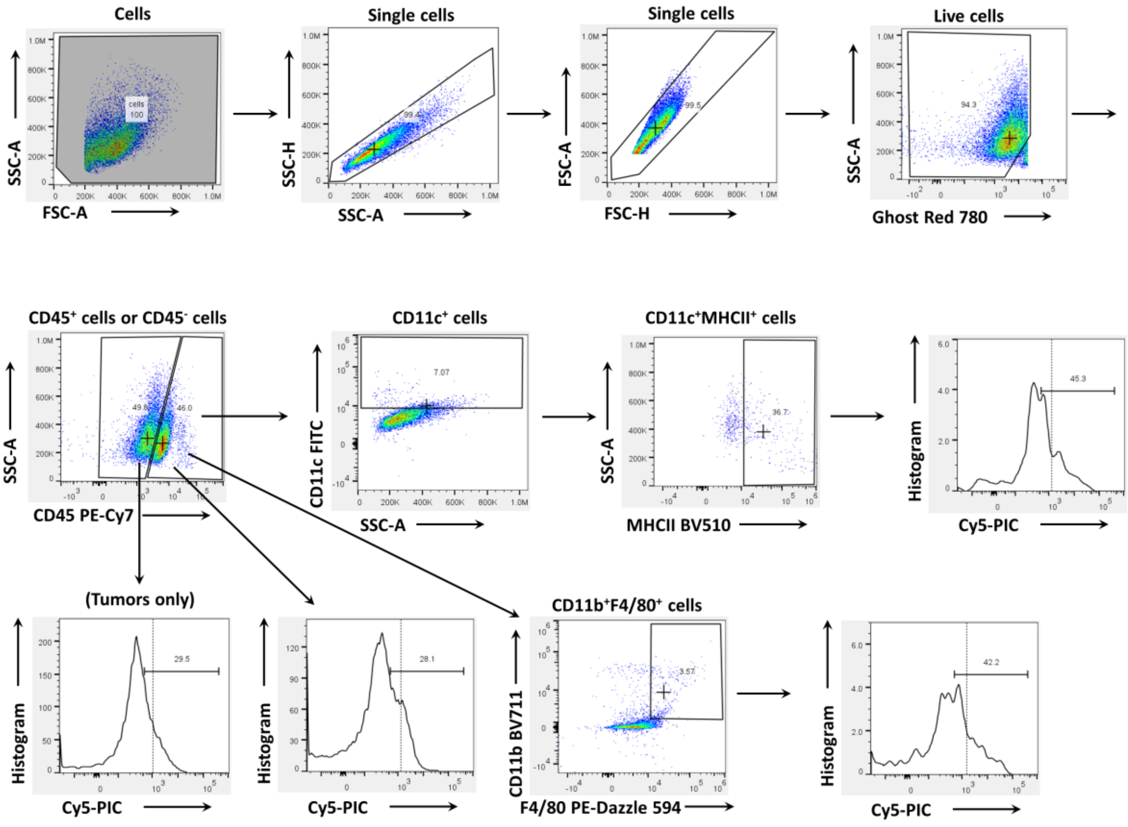

Supplementary Figure 13. Gating strategy for the analysis of Cy5-PIC internalization in antigen presenting cells in the tumor microenvironment and tumor-draining lymph nodes (TDLNs) after it was intratumorally injected into B78 melanoma-bearing mice.

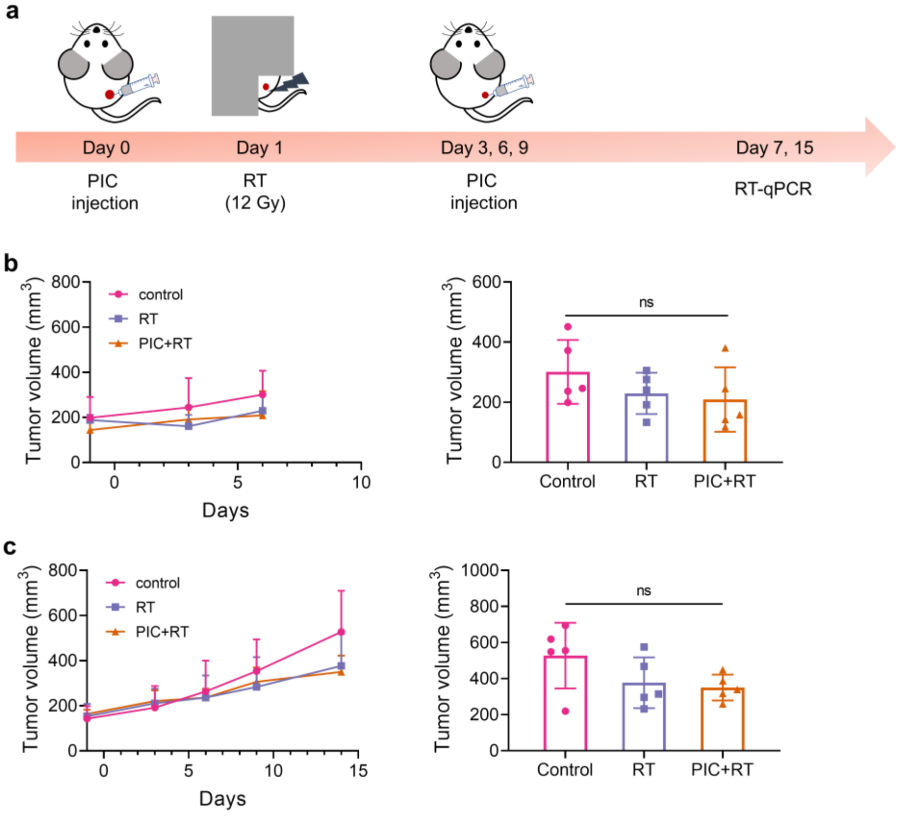

Supplementary Figure 14. (**a**) Scheme for the treatment. The tumor growth curves and average tumor volumes of B78 melanoma bearing mice at (**b**) day 6 and (**c**) day 14 after indicated treatments. The mice were euthanized at (**b**) day 7 and (**c**) day 15 for qPCR analysis of bulk tumor samples. (n=5 biologically independent animals). PIC: 140μg/100μL/dose. RT: 12 Gy. Data are shown as mean ± SD. Statistical significance was calculated via one-way ANOVA test in **b** and **c**. *p<0.05, **p<0.01, ***p<0.001 and ****p<0.0001. Source data are provided in Source Data file.

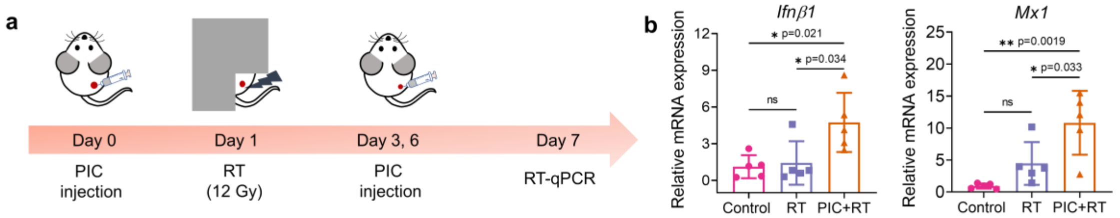

Supplementary Figure 15. (**a**) Scheme for the study of the immunomodulatory effect of PIC+RT on the tumor microenvironment. (**b**) The mRNA expression of *Ifnβ1* and *Mx1* in the B78 tumor microenvironment at day 7 after the indicated treatment. (n=5 biologically independent samples). PIC: 140μg/100μL/dose. RT: 12 Gy. Statistical significance was calculated via one-way ANOVA test in **b** and data are shown as mean ± SD. *p<0.05, **p<0.01, ***p<0.001 and ****p<0.0001. Source data are provided in Source Data file.

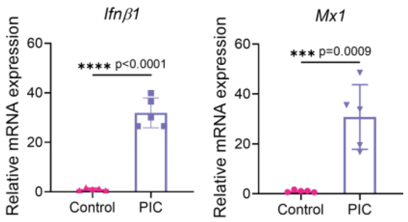

Supplementary Figure 16. The mRNA expression of *Ifnβ1* and *Mx1* in the B78 tumors at day 15 after PIC or control treatment. PIC: 140μg/100μL/dose. PIC was intratumorally injected at day 0, 3, 6 and 9. (n=5 biologically independent samples). Data are shown as mean ± SD. Statistical significance was calculated via unpaired t-test. *p<0.05, **p<0.01, ***p<0.001 and ****p<0.0001. Source data are provided in Source Data file.

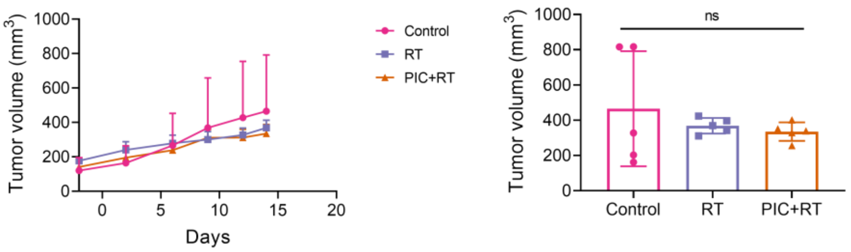

Supplementary Figure 17. The growth of B78 melanoma tumors in syngeneic mice (left) and average tumor volumes at day 14 (right) after indicated treatments. The mice were euthanized at day 15 for flow cytometry analyses of tumors and TDLNs. PIC: 140μg/100μL/dose; RT: 12 Gy. (n=5 biologically independent animals). The indicated treatments were given per **Figure 4a**. Data are shown as mean ± SD. Statistical significance was calculated via one-way ANOVA test. *p<0.05, **p<0.01, ***p<0.001 and ****p<0.0001. Source data are provided in Source Data file.

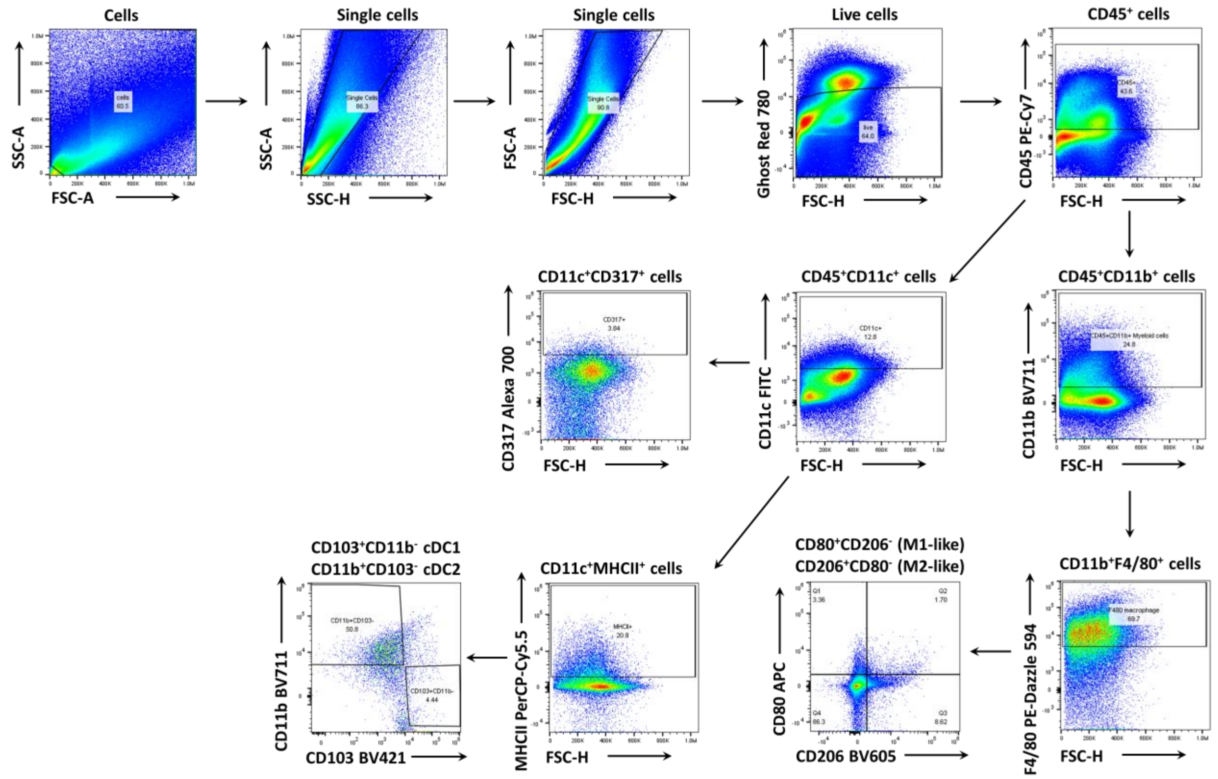

Supplementary Figure 18. Gating strategy for the analysis of innate immune cells in B78 tumors.

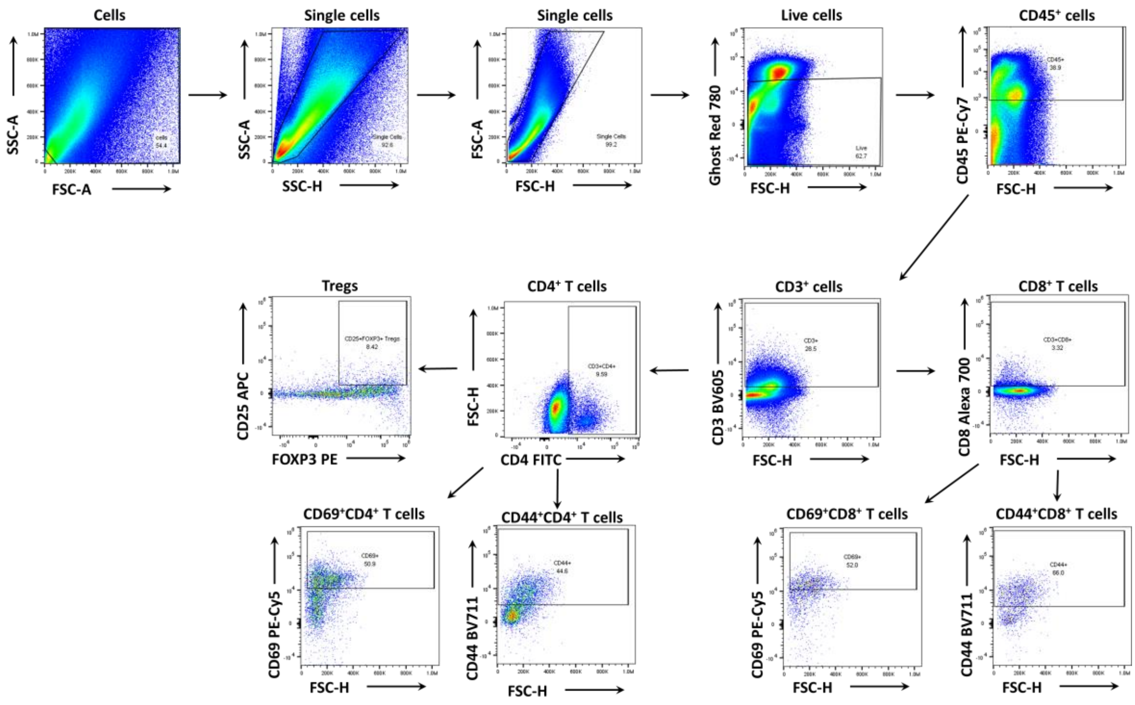

Supplementary Figure 19. Gating strategy for the analysis of adaptive immune cells in B78 tumors.

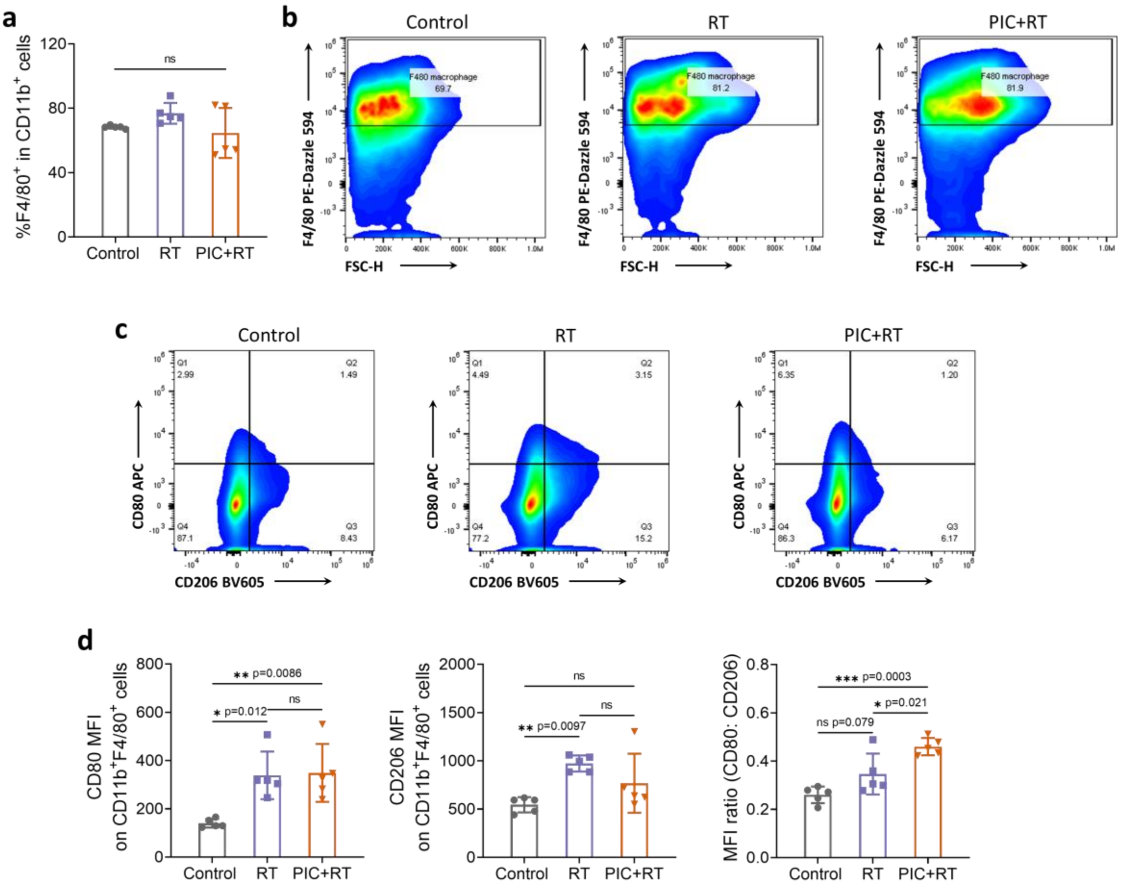

Supplementary Figure 20. (**a**) Quantification and (**b**) representative flow cytometry data identifying F4/80$^+$ cells in CD11b$^+$CD45$^+$ myeloid cells in B78 tumors after indicated treatments. (**c**) Representative flow cytometry data identifying CD80$^+$CD206$^-$ cells (M1-like macrophages) and CD206$^+$CD80$^-$ cells (M2-like macrophages) in CD11b$^+$F4/80$^+$ macrophages in B78 tumors after indicated treatments. (**d**) The mean fluorescence intensity (MFI) of CD80 and CD206, and their ratios relative to total CD11b$^+$F4/80$^+$ macrophages in B78 tumors after indicated treatments. PIC: 140μg/100μL/dose; RT: 12 Gy. (n=5 biologically independent samples). The indicated treatments were given per **Figure 4a** and the tumor samples were collected at day 15 after initiation of indicated treatments. Statistical significance was calculated via one-way ANOVA test in **a** and **d**, and data are shown as mean ± SD. *p<0.05, **p<0.01, ***p<0.001 and ****p<0.0001. Source data are provided in Source Data file.

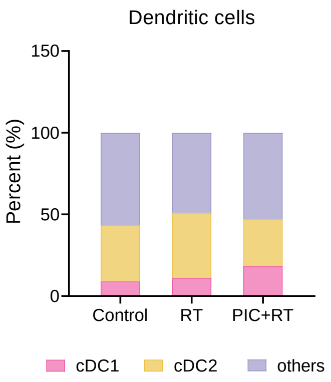

Supplementary Figure 21. The percentage of CD103$^+$CD11b$^-$ cDC1s and CD11b$^+$CD103$^-$ cDC2s among CD11c$^+$MHCII$^+$ DCs in TDLNs from mice bearing B78 flank tumors, after indicated treatments. PIC: 140μg/100μL/dose; RT: 12 Gy. The indicated treatments were given per **Figure 4a** and the tumor samples were collected at day 15 after initiation of indicated treatments. Source data are provided in Source Data file.

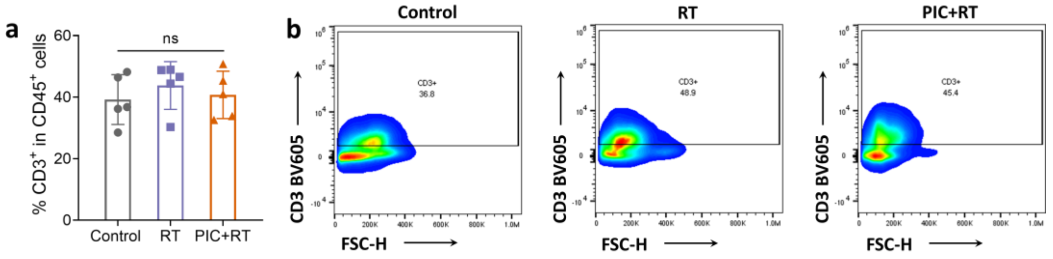

Supplementary Figure 22. (**a**) Quantification and (**b**) representative flow cytometry data identifying CD3$^+$ cells among CD45$^+$ cells in B78 tumors after indicated treatments. PIC: 140μg/100μL/dose; RT: 12 Gy. (n=5 biologically independent samples). The indicated treatments were given per **Figure 4a** and the tumor samples were collected at day 15 after initiation of indicated treatments. Statistical significance was calculated via one-way ANOVA test in **a**, and data are shown as mean ± SD. *p<0.05, **p<0.01, ***p<0.001 and ****p<0.0001. Source data are provided in Source Data file.

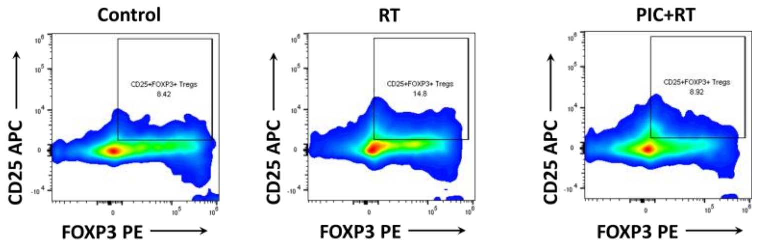

Supplementary Figure 23. Representative flow cytometry data quantifying CD25$^+$FOXP3$^+$ Tregs among CD4$^+$CD3$^+$CD45$^+$ cells in B78 tumors after indicated treatments. PIC: 140μg/100μL/dose; RT: 12 Gy. The indicated treatments were given per **Figure 4a** and the tumor samples were collected at day 15 after

initiation of indicated treatments.

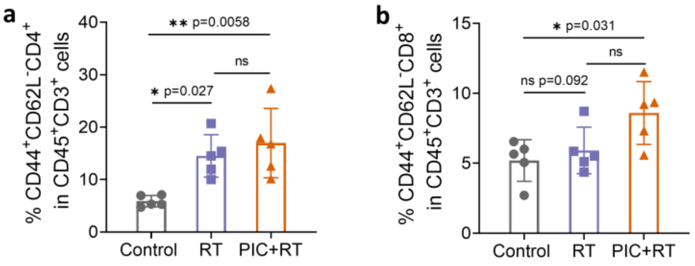

Supplementary Figure 24. The percentage of (**a**) CD44$^+$CD62L$^-$CD4$^+$ and (**b**) CD44$^+$CD62L$^-$CD8$^+$ effector memory cells out of CD45$^+$CD3$^+$ cells in B78 tumors after indicated treatment. PIC: 140μg/100μL/dose; RT: 12 Gy. (n=5 biologically independent samples). The indicated treatments were given per **Figure 4a** and the tumor samples were collected at day 15 after initiation of indicated treatments. Data are shown as mean ± SD. Statistical significance was calculated via one-way ANOVA test. *p<0.05, **p<0.01, ***p<0.001 and ****p<0.0001. Source data are provided in Source Data file.

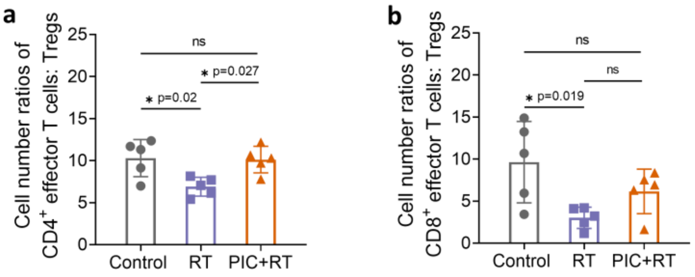

Supplementary Figure 25. The cell number ratios of (**a**) CD4$^+$ effector T cells: Tregs and (**b**) CD8$^+$ effector T cells: Tregs in B78 tumors after indicated treatments. CD4$^+$ effector T cells: CD44$^+$CD4$^+$CD3$^+$CD45$^+$; CD8$^+$ effector T cells: CD44$^+$CD8$^+$CD3$^+$CD45$^+$; Tregs: CD25$^+$FOXP3$^+$CD4$^+$CD3$^+$CD45$^+$. PIC: 140μg/100μL/dose; RT: 12 Gy. (n=5 biologically independent samples). The indicated treatments were given per **Figure 4a** and the tumor samples were collected at day 15 after initiation of indicated treatments. Data are shown as mean ± SD. Statistical significance was calculated via one-way ANOVA test. *p<0.05, **p<0.01, ***p<0.001 and ****p<0.0001. Source data are provided in Source Data file.

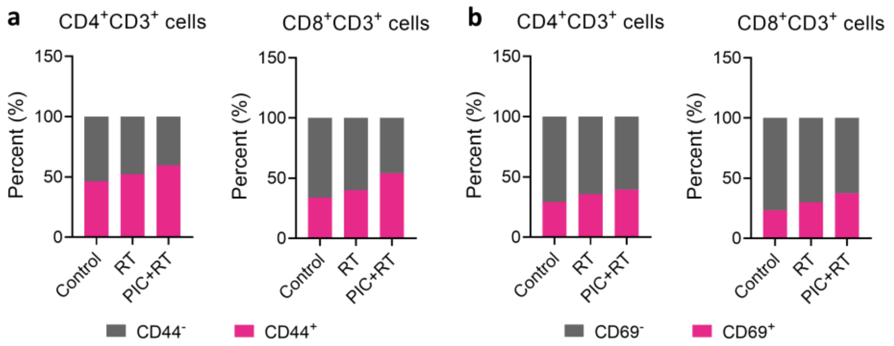

Supplementary Figure 26. The percentage of (**a**) $CD44^+$ and (**b**) $CD69^+$ cells out of $CD3^+CD4^+$ T cells (left) and $CD3^+CD8^+$ T cells (right) in TDLNs after indicated treatments. PIC: 140μg/100μL/dose; RT: 12 Gy. (n=5 biologically independent samples). The indicated treatments were given per **Figure 4a** and the TDLNs were collected at day 15 after initiation of indicated treatments. Source data are provided in Source Data file.

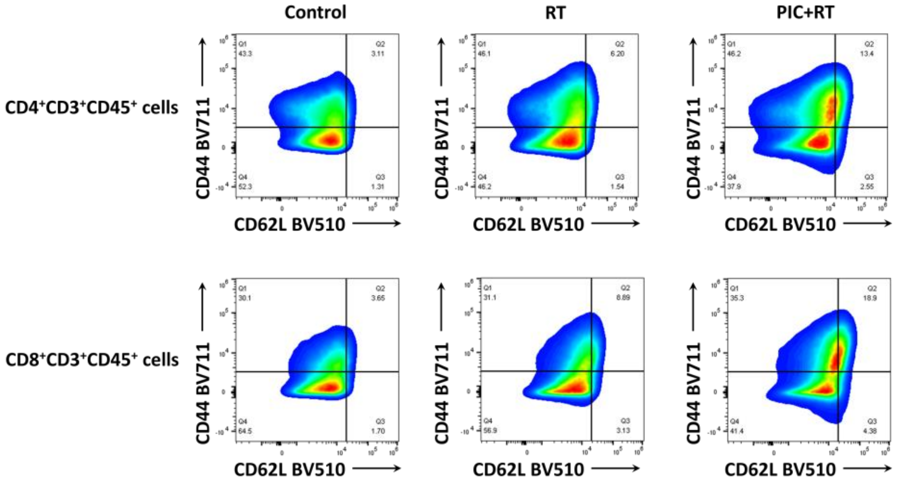

Supplementary Figure 27. Representative flow cytometry data of central memory T cells ($CD44^+CD62L^+$) and effector memory T cells ($CD44^+CD62L^-$) in $CD4^+CD3^+CD45^+$ or $CD8^+CD3^+CD45^+$ cells in TDLNs after indicated treatments. PIC: 140μg/100μL/dose; RT: 12 Gy. The indicated treatments were given per **Figure 4a** and the TDLNs were collected at day 15 after initiation of indicated treatments.

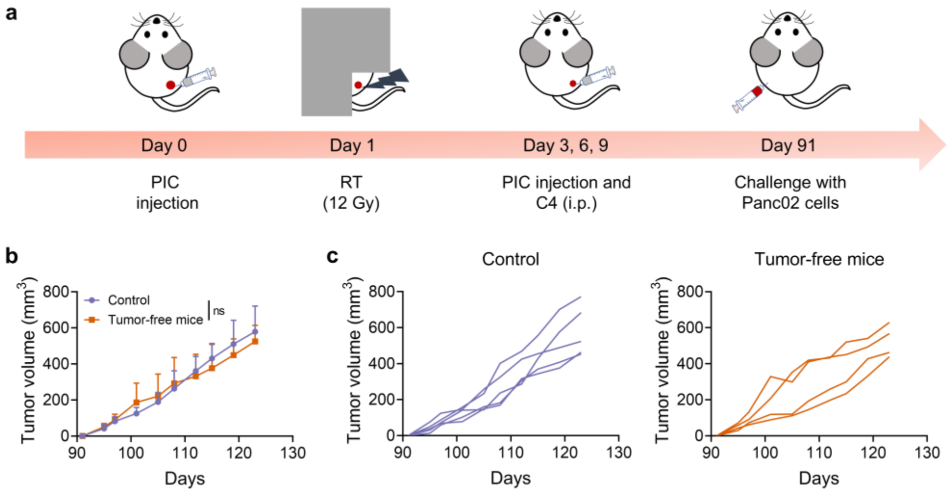

Supplementary Figure 28. (**a**) Scheme for the treatment of mice bearing a B78 melanoma and subsequent implantation with an unrelated Panc02 tumor. (**b**) Average Panc02 tumor growth curves are shown after these tumors were engrafted in naïve control mice or in mice rendered disease-free from a B78 melanoma by PIC+RT+anti-CTLA-4. (control: n=5; tumor-free mice: n=4 biologically independent animals). (**c**) Individual mouse tumor growth curves from (**b**). Data are shown as mean ± SD. Statistical significance was calculated via linear mixed effects modeling in **b**. *p<0.05, **p<0.01, ***p<0.001 and ****p<0.0001. Source data are provided in Source Data file.

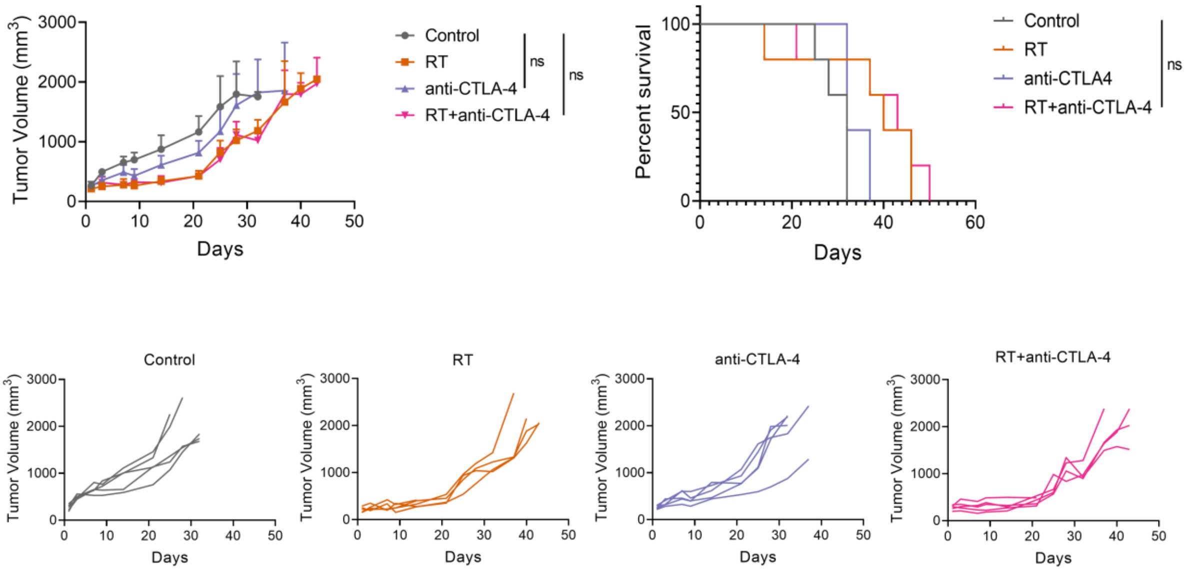

Supplementary Figure 29. TC11 tumor growth and mice survival after indicated treatments. RT: 12 Gy. Anti-CTLA-4: 100 μg/100μL/dose. (n=5 biologically independent animals). The indicated treatments were given per **Figure 6a**. Data are shown as mean ± SD. Statistical significance was calculated via linear mixed effects modeling and log-rank test for tumor growth and mice survival, respectively. *p<0.05, **p<0.01, ***p<0.001 and ****p<0.0001. Source data are provided in Source Data file.

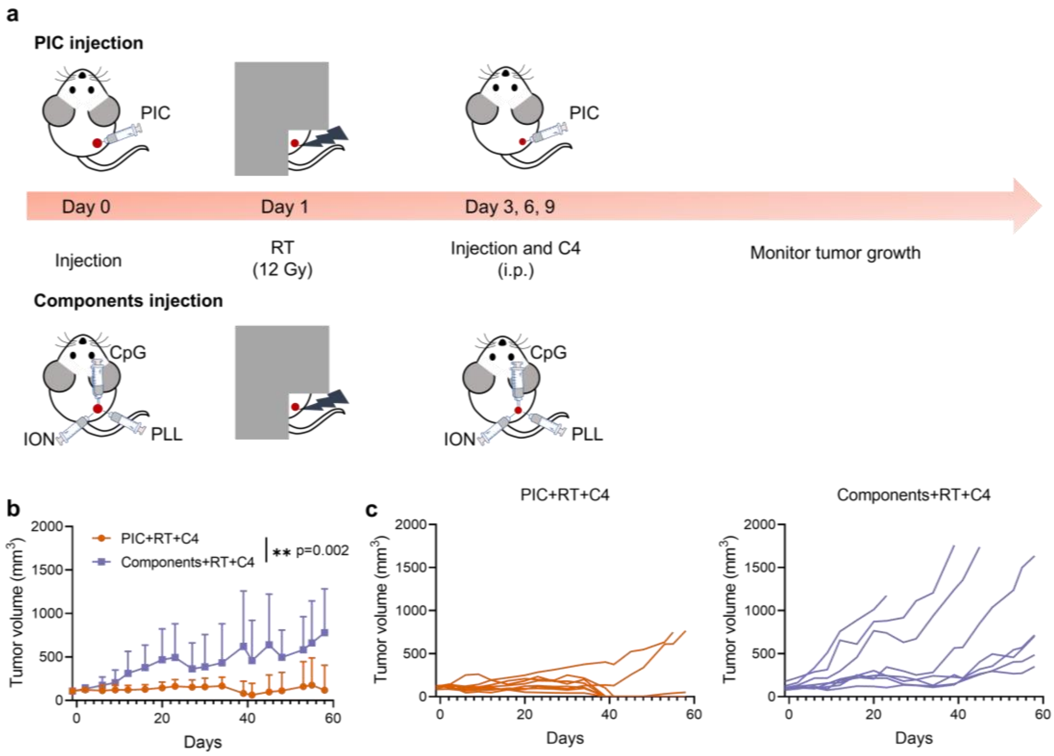

Supplementary Figure 30. (**a**) Scheme for the treatment of mice bearing a B78 melanoma flank tumor. (**b**) Average tumor growth curves of mice are displayed following the indicated treatment regimen. (n=8 biologically independent animals). (**c**) Individual tumor growth curves for mice in (**b**). RT: 12 Gy. PIC: 140 µg/100µL/dose. C4 (anti-CTLA-4): 100 µg/100µL/dose. Statistical significance was calculated via linear mixed effects modeling in **b**, and data are shown as mean ± SD. *p<0.05, **p<0.01, ***p<0.001 and ****p<0.0001. Source data are provided in Source Data file.

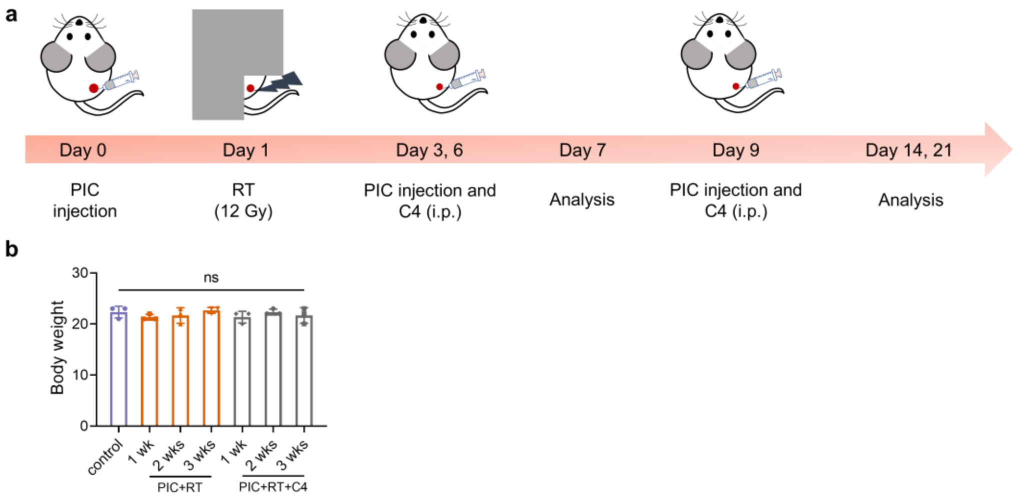

Supplementary Figure 31. (**a**) Scheme for delivery of treatment for in vivo toxicity studies. (**b**) No significant

change was observed in the body weight of mice bearing a B78 melanoma after treatment with PIC + RT or PIC+RT+C4. PIC: 140μg/100μL/dose. RT: 12 Gy. C4 (anti-CTLA-4): 100μg/100μL/dose. (n=3 biologically independent animals). Data are shown as mean ± SD. Statistical significance was calculated via one-way ANOVA test. *p<0.05, **p<0.01, ***p<0.001 and ****p<0.0001. Source data are provided in Source Data file.

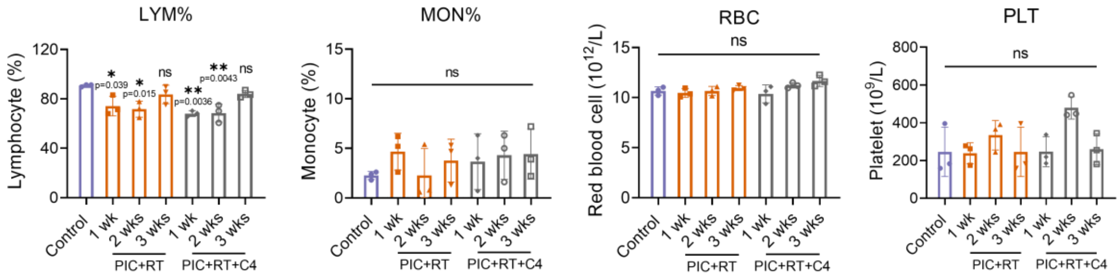

Supplementary Figure 32. The complete blood counts of B78 melanoma bearing mice after indicated treatments. PIC: 140μg/100μL/dose. RT: 12 Gy. C4 (anti-CTLA-4): 100μg/100μL/dose. (n=3 biologically independent samples). The indicated treatments were given per Supplementary Figure 31a. Data are shown as mean ± SD. Statistical significance was calculated via one-way ANOVA test. *p<0.05, **p<0.01, ***p<0.001 and ****p<0.0001. Source data are provided in Source Data file.

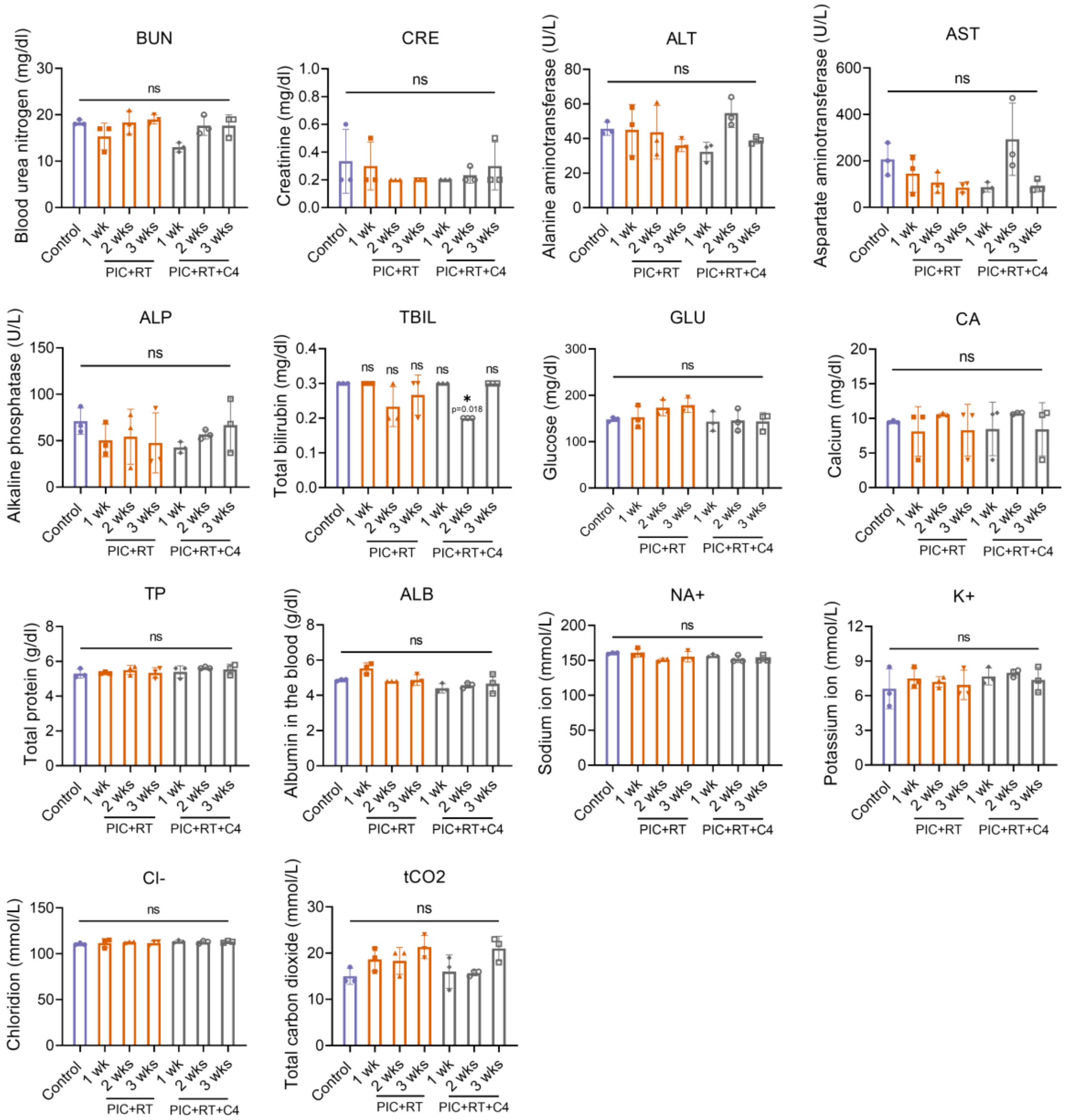

Supplementary Figure 33. The blood metabolic profiles of mice bearing a B78 melanoma after indicated treatments. PIC: 140µg/100µL/dose. RT: 12 Gy. C4 (anti-CTLA-4): 100µg/100µL/dose. (n=3 biologically independent samples). The indicated treatments were given per Supplementary Figure 31a. Data are shown as mean ± SD. Statistical significance was calculated via one-way ANOVA test. *p<0.05, **p<0.01, ***p<0.001 and ****p<0.0001. Source data are provided in Source Data file.

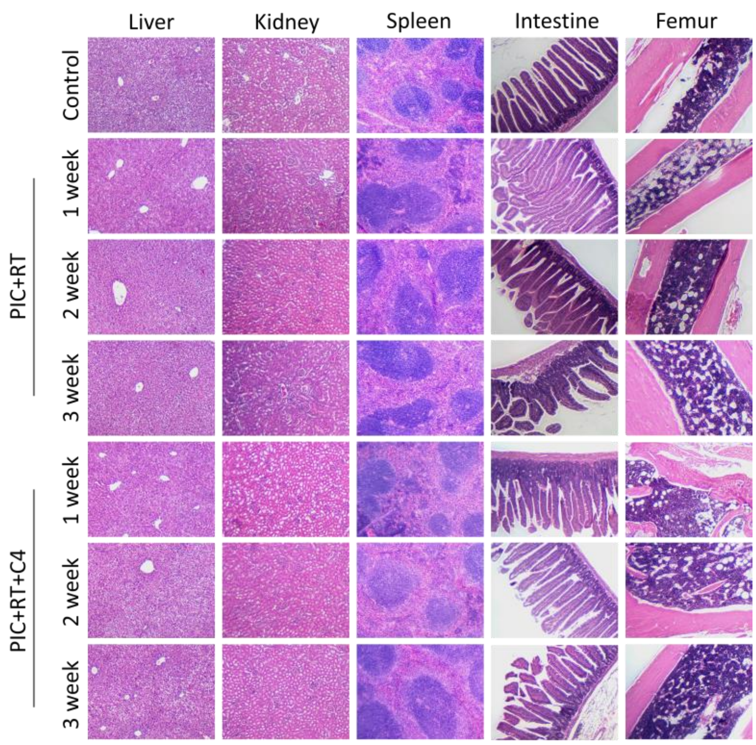

Supplementary Figure 34. Images of H&E stained sections of major organs (liver, spleen, kidney, intestine and femur) from mice bearing a B78 melanoma and treated with PIC+RT or PIC+RT+C4. PIC: 140μg/100μL/dose. RT: 12 Gy. C4 (anti-CTLA-4): 100μg/100μL/dose. The indicated treatments were given per Supplementary Figure 31a. A representative image of three independent samples from each group is shown.

Supplementary Table 1. Particle size and zeta potential of ION/PLL/CpG nanoparticle with different weight ratio.
(w/w/w indicates the weight ratio between different components)

ION/PLL/CpG (w/w/w)	Particle Size (nm)	Zeta potential (mV)
9.6/2/3	141.5nm (PDI: 0.231)	-30.5, -30.5, -29.8
9.6/4/3	precipitation	
9.6/7/3	121.8nm (PDI: 0.127)	24.6, 23.3, 24.4
4.8/3.5/1	113.2nm (PDI: 0.302)	32.8, 34, 34.3

Supplementary Table 2. The cell number data for flow cytometry analyses of innate immune cells from B78 tumors. (The figures are shown in Figure 4b-4h and Supplementary Figure 20)

Sample labels	Live cells	Myeloid cells	Macrophages	M1 macrophages	M2 macrophages	pDCs	$CD80^+$ pDCs	cDC1	cDC2
Control-1	711242	92795	63274	1895	5332	1265	178	529	3650
Control-2	654187	82500	56437	989	3295	745	79	509	4903
Control-3	630078	68331	47612	1598	4105	1350	211	327	3735
Control-4	665761	77567	51967	1652	2775	960	97	447	4948
Control-5	771599	68742	46944	845	3609	509	42	279	2427
RT-1	594992	137753	106056	4765	16117	6716	901	1222	7189
RT-2	359319	61320	49818	2734	8908	5242	869	541	8413
RT-3	532121	86181	75594	5522	10854	5084	637	726	4725
RT-4	494551	64257	45407	2878	8900	7826	1458	412	4941
RT-5	450643	87702	65644	2786	8995	5043	833	516	11326
PIC+RT-1	427793	78873	43160	2608	3172	2254	413	328	2003
PIC+RT-2	608191	129232	105789	7566	20887	14215	2769	1991	4825
PIC+RT-3	435215	87134	56004	3558	3456	2545	438	413	2448
PIC+RT-4	728356	153189	78271	5455	7856	4303	535	1980	5098
PIC+RT-5	705359	140193	76108	4166	5605	4045	845	555	2925

Myeloid cells: $CD45^+CD11b^+$; Macrophages: $CD45^+CD11b^+F4/80^+$;

M1 macrophages: $CD45^+CD11b^+F4/80^+CD80^+CD206^-$; M2 macrophages: $CD45^+CD11b^+F4/80^+CD206^+CD80^-$;

pDCs: $CD45^+CD11c^+CD317^+$; cDC1: $CD45^+CD11c^+MHCII^+CD103^+CD11b^-$;

cDC2: $CD45^+CD11c^+MHCII^+CD11b^+CD103^-$

Supplementary Table 3. The cell number data for flow cytometry analyses of adaptive immune cells from B78 tumors. (The figures are shown in Figure 4i-4o and Supplementary Figure 22-25)

Sample labels	Live cells	T cells	$CD4^+$ T cells	$CD8^+$ T cells	Tregs	Effector $CD4^+$ T cells	Effector $CD8^+$ T cells	Activated $CD4^+$ T cells	Activated $CD8^+$ T cells
Control-1	575638	64391	6167	2136	520	4750	1778	3142	1111
Control-2	496732	84314	7074	5441	864	6026	5131	3582	3228
Control-3	490462	113835	6700	7709	504	6221	7495	3924	3825
Control-4	525719	130090	6935	7651	560	6335	7409	3576	3461
Control-5	575461	87590	5411	4662	418	4885	4461	3564	2751
RT-1	455784	70180	19881	3905	2753	14873	3171	12686	2360
RT-2	334984	93720	12715	5745	1640	11561	5265	9145	3661
RT-3	460347	128827	14716	8037	1632	13287	6686	10795	5256
RT-4	439941	128199	21171	8738	3143	19276	7658	16424	6361
RT-5	380795	138363	22326	13160	2901	21477	12128	14899	6328
PIC+RT-1	354867	75672	14231	8798	1270	13065	7159	9175	5583
PIC+RT-2	523847	82994	28682	6409	3109	24211	5037	22558	4889
PIC+RT-3	387528	140353	15591	11301	1374	14613	10366	10058	5763
PIC+RT-4	570609	183122	60942	35054	2962	36019	18655	23758	11084
PIC+RT-5	597722	109240	16718	13769	1521	14783	12715	10094	7539

T cells: $CD45^+CD3^+$; $CD4^+$ T cells: $CD45^+CD3^+CD4^+$; $CD8^+$ T cells: $CD45^+CD3^+CD8^+$; Tregs: $CD45^+CD3^+CD4^+CD25^+FOXP3^+$;

Effector $CD4^+$ T cells: $CD45^+CD3^+CD4^+CD44^+$; Effector $CD8^+$ T cells: $CD45^+CD3^+CD8^+CD44^+$;

Activated $CD4^+$ T cells: $CD45^+CD3^+CD4^+CD69^+$; Activated $CD8^+$ T cells: $CD45^+CD3^+CD8^+CD69^+$

Supplementary Table 4. The reference of Taqman genes used for RT-qPCR studies.

Taqman gene	Assay ID
Ifnb1 (interferon beta)	Mm00439552_s1
Mx1 (MX dynamin-like GTPase 1)	Mm00487796_m1
Arg1 (Arginase)	Mm00475988_m1
Nos2 (Nitric oxide synthase 2)	Mm00440502_m1
Ifnγ (interferon gamma)	Mm01168134_m1
Il6 (interleukin 6)	Mm00446190_m1
Tnfα (tumor necrosis factor)	Mm00443258_m1
Il1β (interleukin 1 beta)	Mm00434228_m1
Pd-l1 (CD274 antigen)	Mm03048248_m1
Il10 (interleukin 10)	Mm01288386_m1
Tgfβ*1* (transforming growth factor, beta 1)	Mm01178820_m1
Hprt (hypoxanthine guanine phosphoribosyl transferase)	Mm03024075_m1

Supplementary Table 5. Antibodies and markers used for flow cytometry studies.

Antibody/Marker	Fluorophore	Clone	Dilution	Company	Cat number
Live/dead	GhostRed 780		1:200	Tonbo Biosciences	13-0865-CD206T100
F4/80	PE-Dazzle594	BM8	1:100	Biolegend	123146
CD206	BV421	C068C2	1:100	Biolegend	141717
CD11b	BV711	M1/70	1:100	Biolegend	101241
CD80	APC	16-10A1	1:100	Biolegend	104713
CD163	PE-Cy7	S15049I	1:100	Biolegend	155319
CD16/CD32		S17011E	1:200	Biolegend	156604
CD11c	PerCP-Cy5.5	N418	1:100	Tonbo Biosciences	65-0114-U025
CD80	PE	16-10A1	1:100	Tonbo Biosciences	50-0801-U025
CD86	BV605	GL-1	1:100	Biolegend	105037
CD317	Alexa 700	927	1:100	Biolegend	127037
CD4	FITC	RM4-5	1:100	Biolegend	100510
IFN-γ	PE-Dazzle594	XMG1.2	1:100	Biolegend	505845
CD69	PE-Cy5	H1.2F3	1:100	Biolegend	104509
CD45	PE-Cy7	30-F11	1:100	Biolegend	103114
CD3	BV605	17A2	1:100	Biolegend	100237
CD8a	Alexa 700	53-6.7	1:100	Biolegend	100730
FOXP3	PE	MF-14	1:50	Biolegend	126404
PD-1	BV421	RMP1-30	1:100	Biolegend	109121
CD62L	BV510	MEL-14	1:100	Biolegend	104441
CD44	BV711	IM7	1:100	Biolegend	103057
CD25	APC	3C7	1:100	Biolegend	101910
CD11c	FITC	N418	1:100	Tonbo Biosciences	35-0114-U500
MHCII (I-A/I-E)	PerCP-Cy5.5	M5/114.15.2	1:100	Biolegend	107625
CD103	BV421	2E7	1:100	Biolegend	121422
CD206	BV605	C068C2	1:100	Biolegend	141721
MHCII (I-A/I-E)	BV510	M5/114.15.2	1:100	Biolegend	107635
Granzyme B	PE	QA16A02	1:100	Biolegend	372208
CD3	FITC	17A2	1:100	Biolegend	100203
CD4	BV510	GK1.5	1:100	Biolegend	100449
CD8a	PerCP-Cy5.5	53-6.7	1:100	Biolegend	100734
CD69	BV421	H1.2F3	1:100	Biolegend	104527
Phospho-Histone H2A.X (Ser139) Rabbit mAb			1:400	Cell Signaling Technology	9718S

Supplementary Table 6. p values for each comparison in Figure 5b and 5c, analyzed by linear mixed effects modeling and the log-rank test, respectively.

Tumor growth:

	RT	PIC	C4	RT+C4	PIC+RT	PIC+C4	PIC+RT+C4
Control	0.066	0.929	0.518	<0.001	0.035	0.380	<0.001

	Control	RT	PIC	C4	RT+C4	PIC+RT	PIC+C4
PIC+RT+C4	<0.001	<0.001	<0.001	<0.001	<0.001	<0.001	<0.001

Mice survival:

	RT	PIC	C4	RT+C4	PIC+RT	PIC+C4	PIC+RT+C4
Control	0.005	0.755	0.804	<0.001	0.011	0.272	<0.001

	Control	RT	PIC	C4	RT+C4	PIC+RT	PIC+C4
PIC+RT+C4	<0.001	<0.001	<0.001	<0.001	0.001	<0.001	<0.001
