# Supplementary material for: Multifunctional nanoparticle potentiates the in situ vaccination effect of radiation therapy and enhances response to immune checkpoint blockade
Source: Nat Commun. 2022 Aug 23;13:4948. doi: 10.1038/s41467-022-32645-x (PMC9399096; doi:10.1038/s41467-022-32645-x)
Supplement: Supplementary file 3 — Reporting Summary [file 41467_2022_32645_MOESM3_ESM.pdf]

nature portfolio

Corresponding author(s): Zachary Morris

Last updated by author(s): Jul 17, 2022

Reporting Summary

Nature Portfolio wishes to improve the reproducibility of the work that we publish. This form provides structure for consistency and transparency in reporting. For further information on Nature Portfolio policies, see our Editorial Policies and the Editorial Policy Checklist.

Statistics

For all statistical analyses, confirm that the following items are present in the figure legend, table legend, main text, or Methods section.

n/a	Confirmed	
[ ]	[x]	The exact sample size (*n*) for each experimental group/condition, given as a discrete number and unit of measurement
[ ]	[x]	A statement on whether measurements were taken from distinct samples or whether the same sample was measured repeatedly
[ ]	[x]	The statistical test(s) used AND whether they are one- or two-sided<br>*Only common tests should be described solely by name; describe more complex techniques in the Methods section.*
[ ]	[x]	A description of all covariates tested
[ ]	[x]	A description of any assumptions or corrections, such as tests of normality and adjustment for multiple comparisons
[ ]	[x]	A full description of the statistical parameters including central tendency (e.g. means) or other basic estimates (e.g. regression coefficient) AND variation (e.g. standard deviation) or associated estimates of uncertainty (e.g. confidence intervals)
[ ]	[x]	For null hypothesis testing, the test statistic (e.g. *F*, *t*, *r*) with confidence intervals, effect sizes, degrees of freedom and *P* value noted<br>*Give P values as exact values whenever suitable.*
[x]	[ ]	For Bayesian analysis, information on the choice of priors and Markov chain Monte Carlo settings
[x]	[ ]	For hierarchical and complex designs, identification of the appropriate level for tests and full reporting of outcomes
[x]	[ ]	Estimates of effect sizes (e.g. Cohen's *d*, Pearson's *r*), indicating how they were calculated

Our web collection on statistics for biologists contains articles on many of the points above.

Software and code

Policy information about availability of computer code

Data collection	BD FACSDiva (version 8.0.2) and FlowJo (v10) were used to acquire and analyze the flow cytometry data. NIS elements (Nikon) was used to collect the CLSM images. DLS Software (Zetasizer, version 7.01) was used for size and zeta potential measurement. QuantStudio Software v1.3 was used to collect qPCR raw data, and Excel (Office 365) was used for the analysis. Spectrum Living Image 4.0 Software was used to collect the in vivo imaging data. Plates were read by Progmega GloMax.
Data analysis	Prism 8 (GraphPad Software) and R (v 4.0.5) were used for all statistical analyses.

For manuscripts utilizing custom algorithms or software that are central to the research but not yet described in published literature, software must be made available to editors and reviewers. We strongly encourage code deposition in a community repository (e.g. GitHub). See the Nature Portfolio guidelines for submitting code & software for further information.

Data

Policy information about availability of data

All manuscripts must include a data availability statement. This statement should provide the following information, where applicable:

- Accession codes, unique identifiers, or web links for publicly available datasets
- A description of any restrictions on data availability
- For clinical datasets or third party data, please ensure that the statement adheres to our policy

Data availability: All data reported in this work are available within the Article, Supplementary Information, or Source Data file. Source data are provided with this

paper.

Human research participants

Policy information about studies involving human research participants and Sex and Gender in Research.

Reporting on sex and gender	n/a
Population characteristics	n/a
Recruitment	n/a
Ethics oversight	n/a

Note that full information on the approval of the study protocol must also be provided in the manuscript.

Field-specific reporting

Please select the one below that is the best fit for your research. If you are not sure, read the appropriate sections before making your selection.

[x] Life sciences [ ] Behavioural & social sciences [ ] Ecological, evolutionary & environmental sciences

For a reference copy of the document with all sections, see nature.com/documents/nr-reporting-summary-flat.pdf

Life sciences study design

All studies must disclose on these points even when the disclosure is negative.

Sample size	Sample sizes for each experiment are stated in Figure legends and Methods. Sample sizes are determined empirically, and similar in size to most existing studies in the same field (PMID: 27197149, 33995649, 31523868). No method was used to predetermine the sample size.
Data exclusions	No data were excluded.
Replication	The in vitro studies were repeated independently at least twice with similar results. Animal studies were conducted independently twice with similar results.
Randomization	Throughout the whole experiment, samples and animals were randomized into groups.
Blinding	Blinding was not performed for treatments however personnel monitoring tumor growth/response were blinded to treatment groupings during measurements. All samples were treated in the same manner for each experiment. During tumor measurement and toxicity assessment, observers were blinded to treatment groups. Due to technical, safety, and personnel limitations we are not able to blind the delivery of radiotherapy and therefore we did not blind the administration of treatments.

Behavioural & social sciences study design

All studies must disclose on these points even when the disclosure is negative.

Study description	*Briefly describe the study type including whether data are quantitative, qualitative, or mixed-methods (e.g. qualitative cross-sectional, quantitative experimental, mixed-methods case study).*
Research sample	*State the research sample (e.g. Harvard university undergraduates, villagers in rural India) and provide relevant demographic information (e.g. age, sex) and indicate whether the sample is representative. Provide a rationale for the study sample chosen. For studies involving existing datasets, please describe the dataset and source.*
Sampling strategy	*Describe the sampling procedure (e.g. random, snowball, stratified, convenience). Describe the statistical methods that were used to predetermine sample size OR if no sample-size calculation was performed, describe how sample sizes were chosen and provide a rationale for why these sample sizes are sufficient. For qualitative data, please indicate whether data saturation was considered, and what criteria were used to decide that no further sampling was needed.*
Data collection	*Provide details about the data collection procedure, including the instruments or devices used to record the data (e.g. pen and paper, computer, eye tracker, video or audio equipment) whether anyone was present besides the participant(s) and the researcher, and whether the researcher was blind to experimental condition and/or the study hypothesis during data collection.*

Timing	*Indicate the start and stop dates of data collection. If there is a gap between collection periods, state the dates for each sample cohort.*
Data exclusions	*If no data were excluded from the analyses, state so OR if data were excluded, provide the exact number of exclusions and the rationale behind them, indicating whether exclusion criteria were pre-established.*
Non-participation	*State how many participants dropped out/declined participation and the reason(s) given OR provide response rate OR state that no participants dropped out/declined participation.*
Randomization	*If participants were not allocated into experimental groups, state so OR describe how participants were allocated to groups, and if allocation was not random, describe how covariates were controlled.*

Ecological, evolutionary & environmental sciences study design

All studies must disclose on these points even when the disclosure is negative.

Study description	*Briefly describe the study. For quantitative data include treatment factors and interactions, design structure (e.g. factorial, nested, hierarchical), nature and number of experimental units and replicates.*
Research sample	*Describe the research sample (e.g. a group of tagged Passer domesticus, all Stenocereus thurberi within Organ Pipe Cactus National Monument), and provide a rationale for the sample choice. When relevant, describe the organism taxa, source, sex, age range and any manipulations. State what population the sample is meant to represent when applicable. For studies involving existing datasets, describe the data and its source.*
Sampling strategy	*Note the sampling procedure. Describe the statistical methods that were used to predetermine sample size OR if no sample-size calculation was performed, describe how sample sizes were chosen and provide a rationale for why these sample sizes are sufficient.*
Data collection	*Describe the data collection procedure, including who recorded the data and how.*
Timing and spatial scale	*Indicate the start and stop dates of data collection, noting the frequency and periodicity of sampling and providing a rationale for these choices. If there is a gap between collection periods, state the dates for each sample cohort. Specify the spatial scale from which the data are taken*
Data exclusions	*If no data were excluded from the analyses, state so OR if data were excluded, describe the exclusions and the rationale behind them, indicating whether exclusion criteria were pre-established.*
Reproducibility	*Describe the measures taken to verify the reproducibility of experimental findings. For each experiment, note whether any attempts to repeat the experiment failed OR state that all attempts to repeat the experiment were successful.*
Randomization	*Describe how samples/organisms/participants were allocated into groups. If allocation was not random, describe how covariates were controlled. If this is not relevant to your study, explain why.*
Blinding	*Describe the extent of blinding used during data acquisition and analysis. If blinding was not possible, describe why OR explain why blinding was not relevant to your study.*

Did the study involve field work? ☐ Yes ☐ No

Field work, collection and transport

Field conditions	*Describe the study conditions for field work, providing relevant parameters (e.g. temperature, rainfall).*
Location	*State the location of the sampling or experiment, providing relevant parameters (e.g. latitude and longitude, elevation, water depth).*
Access & import/export	*Describe the efforts you have made to access habitats and to collect and import/export your samples in a responsible manner and in compliance with local, national and international laws, noting any permits that were obtained (give the name of the issuing authority, the date of issue, and any identifying information).*
Disturbance	*Describe any disturbance caused by the study and how it was minimized.*

Reporting for specific materials, systems and methods

We require information from authors about some types of materials, experimental systems and methods used in many studies. Here, indicate whether each material, system or method listed is relevant to your study. If you are not sure if a list item applies to your research, read the appropriate section before selecting a response.

Materials & experimental systems

n/a	Involved in the study
☐	☒ Antibodies
☐	☒ Eukaryotic cell lines
☒	☐ Palaeontology and archaeology
☐	☒ Animals and other organisms
☒	☐ Clinical data
☒	☐ Dual use research of concern

Methods

n/a	Involved in the study
☒	☐ ChIP-seq
☐	☒ Flow cytometry
☒	☐ MRI-based neuroimaging

Antibodies

Antibodies used

Live/dead GhostRed 780 (TONBO biosciences, Cat: 13-0865-CD206T100)
F4/80 PE-Dazzle594 (Clone: BM8, Biolegend, Cat: 123146)
CD206 BV421 (Clone: C068C2, Biolegend, Cat: 141717)
CD11b BV711 (Clone: M1/70, Biolegend, Cat: 101241)
CD80 APC (Clone: 16-10A1, Biolegend, Cat: 104713)
CD163 PE-Cy7 (Clone: S15049I, Biolegend, Cat: 155319)
CD11c PerCP-Cy5.5 (Clone: N418, Tonbo Biosciences, 65-0114-U025)
CD80 PE (Clone: 16-10A1, Tonbo Biosciences, 50-0801-U025)
CD86 BV605 (Clone: GL-1, Biolegend, Cat: 105037)
CD317 Alexa 700 (Clone: 927, Biolegend, Cat: 127037)
CD4 FITC (Clone: RM4-5, Biolegend, Cat: 100510)
IFN-gamma PE-Dazzle594 (Clone: XMG1.2, Biolegend, Cat: 505845)
CD69 PE-Cy5 (Clone: H1.2F3, Biolegend, Cat: 104509)
CD45 PE-Cy7 (Clone: 30-F11, Biolegend, Cat: 103114)
CD3 BV605 (Clone: 17A2, Biolegend, Cat: 100237)
CD8a Alexa 700 (Clone: 53-6.7, Biolegend, Cat: 100730)
FOXP3 PE (Clone: MF-14, Biolegend, Cat: 126404)
PD-1 BV421 (Clone: RMP1-30, Biolegend, Cat: 109121)
CD62L BV510 (Clone: MEL-14, Biolegend, Cat: 104441)
CD44 BV711 (Clone: IM7, Biolegend, Cat: 103057)
CD25 APC (Clone: 3C7, Biolegend, Cat: 101910)
CD11c FITC (Clone: N418, Tonbo Biosciences, Cat: 35-0114-U500)
MHCII (I-A/I-E) PerCP-Cy5.5 (Clone: M5/114.15.2, Biolegend, Cat: 107625)
CD103 BV421 (Clone: 2E7, Biolegend, Cat: 121422)
CD206 BV605 (Clone: C068C2, Biolegend, Cat: 141721)
MHCII (I-A/I-E) BV510 (Clone: M5/114.15.2, Biolegend, Cat: 107635)
Granzyme B PE (Clone: QA16A02, Biolegend, Cat: 372208)
CD3 FITC (Clone: 17A2, Biolegend, Cat: 100203)
CD4 BV510 (Clone: GK1.5, Biolegend, Cat: 100449)
CD8a PerCP-Cy5.5 (Clone: 53-6.7, Biolegend, Cat: 100734)
CD69 BV421 (Clone: H1.2F3, Biolegend, Cat: 104527)
CD16/CD32 (Fc block) (Clone: S17011E, BioLegend, Cat: 156604)
Phospho-Histone H2A.X (Ser139) Rabbit mAb (Cell Signaling Technology, Cat: 9718S)

Validation

All antibodies are commercially available and validated by manufacturer's websites for each respective antibody.

Live/dead GhostRed 780: flow cytometry application, PMID: 35452291, 35083098, 35083342.
https://tonbobio.com/products/ghost-dye-red-780

F4/80 PE-Dazzle594: flow cytometry application, PMID: 23554311, 23554311, 25768281,
https://www.biolegend.com/en-us/products/pe-dazzle-594-anti-mouse-f4-80-antibody-10262

CD206 BV421: flow cytometry application, PMID: 22142849, 25991856, 35022622,
https://www.biolegend.com/en-us/products/brilliant-violet-421-anti-mouse-cd206-mmr-antibody-8638

CD11b BV711: flow cytometry application, PMID: 25964477, 26808628, 33046212,
https://www.biolegend.com/en-us/products/brilliant-violet-711-anti-mouse-human-cd11b-antibody-7927

CD80 APC: flow cytometry application, PMID: 23754785, 22308386, 27053762,
https://www.biolegend.com/en-us/products/apc-anti-mouse-cd80-antibody-2340

CD163 PE-Cy7: flow cytometry application, https://www.biolegend.com/en-us/products/pe-cyanine7-anti-mouse-cd163-antibody-20615

CD11c PerCP-Cy5.5: flow cytometry application, PMID: 21300822, 21307334.
https://tonbobio.com/products/percp-cyanine5-5-anti-mouse-cd11c-n418

CD80 PE: flow cytometry application, PMID: 22745171, 20548032,
https://tonbobio.com/products/pe-anti-mouse-cd80-b7-1-16-10a1

CD86 BV605: flow cytometry application, PMID: 24123688, 25888644, 32692155
https://www.biolegend.com/en-us/products/brilliant-violet-605-anti-mouse-cd86-antibody-7798

CD317 Alexa 700: flow cytometry application, PMID: 16920966, 19903902, 24145513,
https://www.biolegend.com/en-us/products/alexa-fluor-700-anti-mouse-cd317-bst2-pdca-1-antibody-18724

CD4 FITC: flow cytometry application, PMID: 23980105, 25114223, 26347472,
https://www.biolegend.com/en-us/products/fitc-anti-mouse-cd4-antibody-480

IFN-gamma PE-Dazzle594: flow cytometry application, PMID: 30174303, 31040289, 28421694,
https://www.biolegend.com/en-us/products/pe-dazzle-594-anti-mouse-ifn-gamma-antibody-9986

CD69 PE-Cy5: flow cytometry application, PMID: 20656015, 31053504, 32702313,
https://www.biolegend.com/en-us/products/pe-cyanine5-anti-mouse-cd69-antibody-266

CD45 PE-Cy7: flow cytometry application, PMID: 22547694, 35022622, 22558218,
https://www.biolegend.com/en-us/products/pe-cyanine7-anti-mouse-cd45-antibody-1903

CD3 BV605: flow cytometry application, PMID: 33376221, 35022622, 33838102,
https://www.biolegend.com/en-us/products/brilliant-violet-605-anti-mouse-cd3-antibody-8503

CD8a Alexa 700: flow cytometry application, PMID: 16116223, 24078696, 27708334,
https://www.biolegend.com/en-us/products/alexa-fluor-700-anti-mouse-cd8a-antibody-3387

FOXP3 PE: flow cytometry application, PMID: 19841163, 22532866, 33826903,
https://www.biolegend.com/en-us/products/pe-anti-mouse-foxp3-antibody-4660

PD-1 BV421: flow cytometry application, PMID: 16493037, 19380638, 32910906
https://www.biolegend.com/en-us/products/brilliant-violet-421-anti-mouse-cd279-pd-1-antibody-14324

CD62L BV510: flow cytometry application, PMID: 17620363, 17702899, 18818390,
https://www.biolegend.com/en-us/products/brilliant-violet-510-anti-mouse-cd62l-antibody-8162

CD44 BV711: flow cytometry application, PMID: 15905539, 18045971, 32516589,
https://www.biolegend.com/en-us/products/brilliant-violet-711-anti-mouse-human-cd44-antibody-10316

CD25 APC: flow cytometry application, PMID: 23182710, 26964093, 30926232,
https://www.biolegend.com/en-us/products/apc-anti-mouse-cd25-antibody-4512

CD11c FITC: flow cytometry application, PMID: 30446384, 30817215,
https://tonbobio.com/products/fitc-anti-mouse-cd11c-n418

MHCII (I-A/I-E) PerCP-Cy5.5: flow cytometry application, PMID: 16973389, 22719068, 25429074,
https://www.biolegend.com/en-us/products/percp-cyanine5-5-anti-mouse-i-a-i-e-antibody-4282

CD103 BV421: flow cytometry application, PMID: 21551361, 26265006, 31932810,
https://www.biolegend.com/en-us/products/brilliant-violet-421-anti-mouse-cd103-antibody-7329

CD206 BV605: flow cytometry application, PMID: 22142849, 33376221, 27277683,
https://www.biolegend.com/en-us/products/brilliant-violet-605-anti-mouse-cd206-mmr-antibody-8729

MHCII (I-A/I-E) BV510: flow cytometry application, PMID: 16973389, 18573901, 26200783,
https://www.biolegend.com/en-us/products/brilliant-violet-510-anti-mouse-i-a-i-e-antibody-7997

Granzyme B PE: flow cytometry application, PMID: 32665441, 32574709, 31442407
https://www.biolegend.com/en-us/products/pe-anti-human-mouse-granzyme-b-recombinant-antibody-14431

CD3 FITC: flow cytometry application, PMID: 33376221, 35022622, 23082146
https://www.biolegend.com/en-us/products/fitc-anti-mouse-cd3-antibody-45

CD4 BV510: flow cytometry application, PMID: 33376221, 33340886, 30568034
https://www.biolegend.com/en-us/products/brilliant-violet-510-anti-mouse-cd4-antibody-10707

CD8a PerCP-Cy5.5: flow cytometry application, PMID: 16263755, 23460738, 16116223, https://www.biolegend.com/en-us/products/percp-cyanine5-5-anti-mouse-cd8a-antibody-4255

CD69 BV421: flow cytometry application, PMID: 18003887, 32445619, 30712876, https://www.biolegend.com/en-us/products/brilliant-violet-421-anti-mouse-cd69-antibody-7358

CD16/CD32 (Fc block): flow cytometry application, PMID: 33216805, 32663200, 32923137, https://www.biolegend.com/en-us/products/trustain-fcx-plus-anti-mouse-cd16-32-antibody-17085?GroupID=GROUP20

Phospho-Histone H2A.X (Ser139) Rabbit mAb: immunofluorescence staining (primary antibody), PMID: 20493860, 19092802, https://www.cellsignal.com/products/primary-antibodies/phospho-histone-h2a-x-ser139-20e3-rabbit-mab/9718?site-search-type=Products&N=4294956287&Ntt=phospho-histone+h2a.x+%28ser139%29+rabbit+mab&fromPage=plp

Eukaryotic cell lines

Policy information about cell lines and Sex and Gender in Research

Cell line source(s)

B78(B78-D14, GD2+) melanoma cells (from Ralph Reisfeld (Scripps Research Institute)), B16 melanoma cells (from Memorial Sloan Kettering Cancer Center), RAW264.7 cells (ATCC: TIB-71), MyC-CaP prostate tumor cells (ATCC, CRL-3255), TC11 breast tumor cells (generated from an ER+ mammary tumor that developed in a NRL-PRL female, PMID: 31406251), Panc02 cells (National Cancer Institute). B16 cells were transduced to express SIINFEKL via lentiviral transduction pLV[Exp]-Hygro-CBh>SIINFEKL (VectorBuilder; VB210327-1014dyd), which is a lentiviral plasmid that we designed using VectorBuilder's platform. Positively transduced cells were referred to as B16-SIINFEKL (a kind gift from Dr. Amy Erbe), and were selected for using hygromycin (50 ug/ml). Stably transduced cells were single-cell cloned. Clones were selected for downstream use following IFN (100 U/mL; cat #505702, Biolegend) stimulation, and screened for MHC-I presentation of SIINFEKL via flow cytometry on an Attune NxT Flow Cytometer (Thermofisher) using anti-mouse H-2Kb bound SIINFEKL-APC (clone 25-D1.16, cat # 141605, Biolegend).

Authentication

Cell authentication was performed per ATCC guidelines using morphology, growth curves and Mycoplasma testing within 6 months of use and routinely thereafter.

Mycoplasma contamination

All cell lines tested negative for contamination with mycoplasma.

Commonly misidentified lines
(See ICLAC register)

No commonly misidentified cell lines were used in this study.

Palaeontology and Archaeology

Specimen provenance

Provide provenance information for specimens and describe permits that were obtained for the work (including the name of the issuing authority, the date of issue, and any identifying information). Permits should encompass collection and, where applicable, export.

Specimen deposition

Indicate where the specimens have been deposited to permit free access by other researchers.

Dating methods

If new dates are provided, describe how they were obtained (e.g. collection, storage, sample pretreatment and measurement), where they were obtained (i.e. lab name), the calibration program and the protocol for quality assurance OR state that no new dates are provided.

☐ Tick this box to confirm that the raw and calibrated dates are available in the paper or in Supplementary Information.

Ethics oversight

Identify the organization(s) that approved or provided guidance on the study protocol, OR state that no ethical approval or guidance was required and explain why not.

Note that full information on the approval of the study protocol must also be provided in the manuscript.

Animals and other research organisms

Policy information about studies involving animals; ARRIVE guidelines recommended for reporting animal research, and Sex and Gender in Research

Laboratory animals

Mice, wild-type C57BL/6 and FVB/NTac, male and female, 7-8 weeks, Taconic.

Wild animals

No wild animals were used in this study.

Reporting on sex

FVB/NTac male mice were used for MyC-CaP prostate cancer model, and FVB/NTac female mice were used for TC11 breast cancer model. Both female and male mice were used for other animal experiments.

Field-collected samples

This study did not involve samples collected from the field.

Ethics oversight	All animal experiments were performed under the Guide for the Care and Use of Laboratory Animals (National Institutes of Health) and protocol (M005670) approved by the Institutional Animal Care and Use Committee (IACUC) at the University of Wisconsin.

Note that full information on the approval of the study protocol must also be provided in the manuscript.

Clinical data

Policy information about clinical studies

All manuscripts should comply with the ICMJEguidelines for publication of clinical research and a completedCONSORT checklist must be included with all submissions.

Clinical trial registration	*Provide the trial registration number from ClinicalTrials.gov or an equivalent agency.*
Study protocol	*Note where the full trial protocol can be accessed OR if not available, explain why.*
Data collection	*Describe the settings and locales of data collection, noting the time periods of recruitment and data collection.*
Outcomes	*Describe how you pre-defined primary and secondary outcome measures and how you assessed these measures.*

Dual use research of concern

Policy information about dual use research of concern

Hazards

Could the accidental, deliberate or reckless misuse of agents or technologies generated in the work, or the application of information presented in the manuscript, pose a threat to:

No	Yes	
☐	☐	Public health
☐	☐	National security
☐	☐	Crops and/or livestock
☐	☐	Ecosystems
☐	☐	Any other significant area

Experiments of concern

Does the work involve any of these experiments of concern:

No	Yes	
☐	☐	Demonstrate how to render a vaccine ineffective
☐	☐	Confer resistance to therapeutically useful antibiotics or antiviral agents
☐	☐	Enhance the virulence of a pathogen or render a nonpathogen virulent
☐	☐	Increase transmissibility of a pathogen
☐	☐	Alter the host range of a pathogen
☐	☐	Enable evasion of diagnostic/detection modalities
☐	☐	Enable the weaponization of a biological agent or toxin
☐	☐	Any other potentially harmful combination of experiments and agents

ChIP-seq

Data deposition

☐ Confirm that both raw and final processed data have been deposited in a public database such as GEO.

☐ Confirm that you have deposited or provided access to graph files (e.g. BED files) for the called peaks.

Data access links<br>*May remain private before publication.*	*For "Initial submission" or "Revised version" documents, provide reviewer access links. For your "Final submission" document, provide a link to the deposited data.*
Files in database submission	*Provide a list of all files available in the database submission.*
Genome browser session<br>(e.g. UCSC)	*Provide a link to an anonymized genome browser session for "Initial submission" and "Revised version" documents only, to enable peer review. Write "no longer applicable" for "Final submission" documents.*

Methodology

Replicates	*Describe the experimental replicates, specifying number, type and replicate agreement.*
Sequencing depth	*Describe the sequencing depth for each experiment, providing the total number of reads, uniquely mapped reads, length of reads and whether they were paired- or single-end.*
Antibodies	*Describe the antibodies used for the ChIP-seq experiments; as applicable, provide supplier name, catalog number, clone name, and lot number.*
Peak calling parameters	*Specify the command line program and parameters used for read mapping and peak calling, including the ChIP, control and index files used.*
Data quality	*Describe the methods used to ensure data quality in full detail, including how many peaks are at FDR 5% and above 5-fold enrichment.*
Software	*Describe the software used to collect and analyze the ChIP-seq data. For custom code that has been deposited into a community repository, provide accession details.*

Flow Cytometry

Plots

Confirm that:

- [x] The axis labels state the marker and fluorochrome used (e.g. CD4-FITC).
- [x] The axis scales are clearly visible. Include numbers along axes only for bottom left plot of group (a 'group' is an analysis of identical markers).
- [x] All plots are contour plots with outliers or pseudocolor plots.
- [x] A numerical value for number of cells or percentage (with statistics) is provided.

Methodology

Sample preparation	Single cells suspensions were generated from cell samples or tumor samples after filtered through a 70 µm cell strainer, and red blood cells were lysed using RBC lysis buffer. After stained by antibodies, the cells were analyzed by flow cytometry. UltraComp Beads eBeads (Invitrogen) were used for compensation. All samples were incubated with CD16/CD32 (Fc block) for 5 minutes at room temperature before staining.
Instrument	Attune Cytometer (ThermoFisher)
Software	Attune NxT Software and FlowJo10
Cell population abundance	No cell sorting was performed.
Gating strategy	Briefly, single cells were selected by FSC and SSC plots. Live cells were selected by Live Dead Staining. Immune cells were gated by CD45+ cells. Gating strategies were indicated in details in Supplementary Figure S16-S17.

- [x] Tick this box to confirm that a figure exemplifying the gating strategy is provided in the Supplementary Information.

Magnetic resonance imaging

Experimental design

Design type	*Indicate task or resting state; event-related or block design.*
Design specifications	*Specify the number of blocks, trials or experimental units per session and/or subject, and specify the length of each trial or block (if trials are blocked) and interval between trials.*
Behavioral performance measures	*State number and/or type of variables recorded (e.g. correct button press, response time) and what statistics were used to establish that the subjects were performing the task as expected (e.g. mean, range, and/or standard deviation across subjects).*

Acquisition

Imaging type(s) | *Specify: functional, structural, diffusion, perfusion.*

Field strength | *Specify in Tesla*

Sequence & imaging parameters | *Specify the pulse sequence type (gradient echo, spin echo, etc.), imaging type (EPI, spiral, etc.), field of view, matrix size, slice thickness, orientation and TE/TR/flip angle.*

Area of acquisition | *State whether a whole brain scan was used OR define the area of acquisition, describing how the region was determined.*

Diffusion MRI ☐ Used ☐ Not used

Preprocessing

Preprocessing software | *Provide detail on software version and revision number and on specific parameters (model/functions, brain extraction, segmentation, smoothing kernel size, etc.).*

Normalization | *If data were normalized/standardized, describe the approach(es): specify linear or non-linear and define image types used for transformation OR indicate that data were not normalized and explain rationale for lack of normalization.*

Normalization template | *Describe the template used for normalization/transformation, specifying subject space or group standardized space (e.g. original Talairach, MNI305, ICBM152) OR indicate that the data were not normalized.*

Noise and artifact removal | *Describe your procedure(s) for artifact and structured noise removal, specifying motion parameters, tissue signals and physiological signals (heart rate, respiration).*

Volume censoring | *Define your software and/or method and criteria for volume censoring, and state the extent of such censoring.*

Statistical modeling & inference

Model type and settings | *Specify type (mass univariate, multivariate, RSA, predictive, etc.) and describe essential details of the model at the first and second levels (e.g. fixed, random or mixed effects; drift or auto-correlation).*

Effect(s) tested | *Define precise effect in terms of the task or stimulus conditions instead of psychological concepts and indicate whether ANOVA or factorial designs were used.*

Specify type of analysis: ☐ Whole brain ☐ ROI-based ☐ Both

Statistic type for inference (See Eklund et al. 2016) | *Specify voxel-wise or cluster-wise and report all relevant parameters for cluster-wise methods.*

Correction | *Describe the type of correction and how it is obtained for multiple comparisons (e.g. FWE, FDR, permutation or Monte Carlo).*

Models & analysis

n/a	Involved in the study
☐	☐ Functional and/or effective connectivity
☐	☐ Graph analysis
☐	☐ Multivariate modeling or predictive analysis

Functional and/or effective connectivity | *Report the measures of dependence used and the model details (e.g. Pearson correlation, partial correlation, mutual information).*

Graph analysis | *Report the dependent variable and connectivity measure, specifying weighted graph or binarized graph, subject- or group-level, and the global and/or node summaries used (e.g. clustering coefficient, efficiency, etc.).*

Multivariate modeling and predictive analysis | *Specify independent variables, features extraction and dimension reduction, model, training and evaluation metrics.*
